# Evolutionary history of a widespread tree species *Acer mono* in East Asia

**DOI:** 10.1002/ece3.1278

**Published:** 2014-10-27

**Authors:** Xi-Di Guo, Hong-Fang Wang, Lei Bao, Tian-Ming Wang, Wei-Ning Bai, Jun-Wei Ye, Jian-Ping Ge

**Affiliations:** State Key Laboratory of Earth Surface Processes and Resource Ecology & College of Life Sciences, Beijing Normal UniversityBeijing, 100875, China

**Keywords:** *Acer mono*, community assemblage, phylogeography, temperate mixed forests

## Abstract

East Asia has the most diverse temperate flora in the world primarily due to the lack of Pleistocene glaciation and the geographic heterogeneity. Although increasing phylogeography studies in this region provided more proofs in this issue, discrepancies and uncertainty still exist, especially in northern temperate deciduous broad-leaved and coniferous mixed forest region (II). And a widespread plant species could reduce the complexity to infer the relationship between diversity and physiographical pattern. Hence, we studied the evolution history of a widespread temperate tree, *Acer mono*, populations in region II and the influence of physiographic patterns on intraspecific genetic diversity. Analyses of chloroplast sequences and nuclear microsatellites indicated high levels of genetic diversity. The diversity distribution was spatially heterogeneous and a latitudinal cline existed in both markers. The spatial distribution pattern between genetic diversity within A. mono and the diversity at species level was generally consistent. Western subtropical evergreen broad-leaved forest subregion (IVb) had a unique ancient chloroplast clade (CP3) and a nuclear gene pool (GP5) with dominance indicating the critical role of this area in species diversification. Genetic data and ecological niche model results both suggested that populations in region II disappeared during the last glacial maximum (LGM) and recovered from south of Changbai Mt. and the Korean Peninsula. Two distribution centers were likely during the LGM, one in the north edge of warm temperate deciduous broad-leaved forest region (III) and another in the south edge of region III. This was reflected by the genetic pattern with two spatially independent genetic groups. This study highlights the key role of region III in sustaining genetic diversity in the northern range and connecting diversity between southern and northern range. We elucidated the diversity relationship between vegetation regions which could facilitate the understanding of biodiversity origin and maintenance in East Asia.

## Introduction

The Sino-Japanese Floristic Region (SJFR) in East Asia is one of the most diverse temperate floras in the world, with approximately twice as many plant species as eastern North America, where holds similar size and environment (Qian 2002). Several hypotheses explain this unusually high diversity. This area was not glaciated during the Quaternary compared to eastern North America and Europe where large land ice caps developed. However, reduced temperatures during glaciation periods can still influence species ranges, especially those in the temperate deciduous broad-leaved and coniferous mixed forest region (II), which is the northern distribution limit of many temperate plant species. Pollen records suggest that these northernmost populations probably retreated south (around 25–30°N) during the last glacial maximum (LGM) (Harrison et al. 2001), and the extant populations originated from expansion after the LGM. If this hypothesis is true, then northernmost populations probably experienced a long distance migration, at least 1000 km. Whereas a few phylogeography studies on temperate tree species in region II have demonstrated that cryptic refugia in situ may exist, such as populations of *Juglans mandshurica* (Bai et al. 2010), *Quercus mongolica* (Zeng et al. 2010), *Fraxinus mandschurica* (Hu et al. 2008). These studies have found unique genetic diversity in northern (>30°N) species ranges (Qiu et al. 2011; Liu et al. 2012). These studies show that the cryptic refugia were not consistent among species. For example, either Changbai Mt. or Taihang Mt. could be the LGM refugia for *J. mandshurica* while the refugia for *F. mandschurica* suggested a even northern refugia (around 40°N). Discrepancies between pollen record and molecular data in region II require additional analysis to accurately locate the northern extent of glacial refugia.

High physiographical heterogeneity is considered to be another major influence on the extreme high floral diversity within the SJFR (Qian and Ricklefs 2000). A component of this heterogeneity is a series of mountains extending from southwest to northeast China, such as Hengduan Mt., Qinling Mt., Taihang Mt., Yanshan Mt., and Changbai Mt.. These mountains, with elevations often above 2000 m, provided diverse habitats allowing for species survival during exceptionally cold periods of climate change. In modern subtropical zones, cool environments at higher elevations are suitable for survival, as relict populations, of many temperate species. These relict populations may have allowed for the divergence between extant populations (Hewitt 1996). A large mountain chain could also significantly alter local climate. This would be especially true for mountains extending east to west blocking the flow of cold air into southern regions. In the East Asia mainland, the boundary of many vegetation regions locates in these mountains (Zhang 2007). Qinling Mt. separates the northern warm temperate deciduous broad-leaved forest region (III) from the southern subtropical evergreen broad-leaved forest region (IV), while the southern Changbai Mt. (Qianshan Mt.) separates region II from region III. Hengduan Mt. which extends from north to south in southwestern China provides a unique environment in western region IV, and hence it is suggested that this be divided into two vegetation subregions (eastern IVa and western IVb). The climate and physiography of these vegetation regions/subregions are heterogeneous. The heterogeneity patterns are correlated to plant species diversity with the southern range (region IV) greater than the northern range (regions III and II) and region IVb greater than region IVa. The degree to which habitat heterogeneity contributes to biodiversity and the relationship between vegetation regions are important aspects of the evolution of uniquely high East Asia plant biodiversity. Previous researchers have made significant contributions to our understanding of the origins and extent of biodiversity among the different vegetation regions (Wu et al. 2008; Wulff 1932). However, considering the complexity of intraspecific and interspecific level, our approach was to study the demography and adaptation history of one species with a widespread distribution which may offer more substantial and clear evidences for these questions.

**Figure 1:**
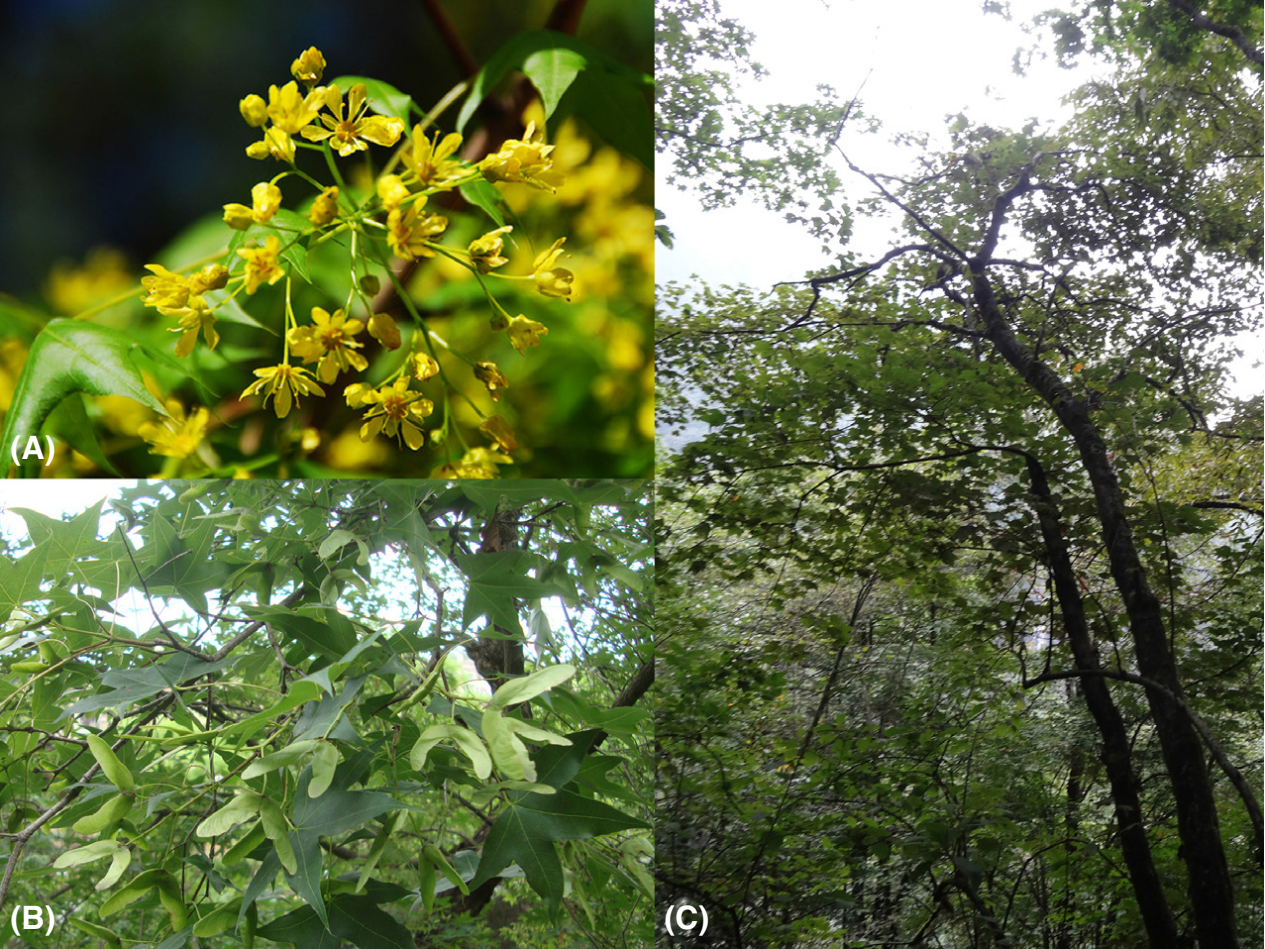
(A) Flowers of *Acer mono*. (B) Samara of *A. mono*. (C) An *A. mono* tree in warm temperate deciduous broad-leaved forest.

*Acer mono* is a widespread tree species common in all vegetation regions within the SJFR. It is a Tertiary relict species (Li 1993) bee or fly pollinated, with wind-mediated seed dispersal (Fig. 1). This is an ideal model species to use for understanding the evolution process in East Asia and the origin and maintenance of biodiversity. We used both nuclear and chloroplast genetic markers and ecological niche modeling (ENM) to answer several questions: (1) Have populations in region II experienced glacial contraction and postglacial expansion? If so, where were the possible locations sustaining relict populations during the LGM? (2) Did the intraspecific genetic diversity pattern correlate to floristic divisions in East Asia, that is, did southern populations hold more genetic diversity than northern populations? Similarly, does the western population harbor higher primitive genetic diversity than eastern range?

## Materials and Methods

### Sampling

**Figure 2:**
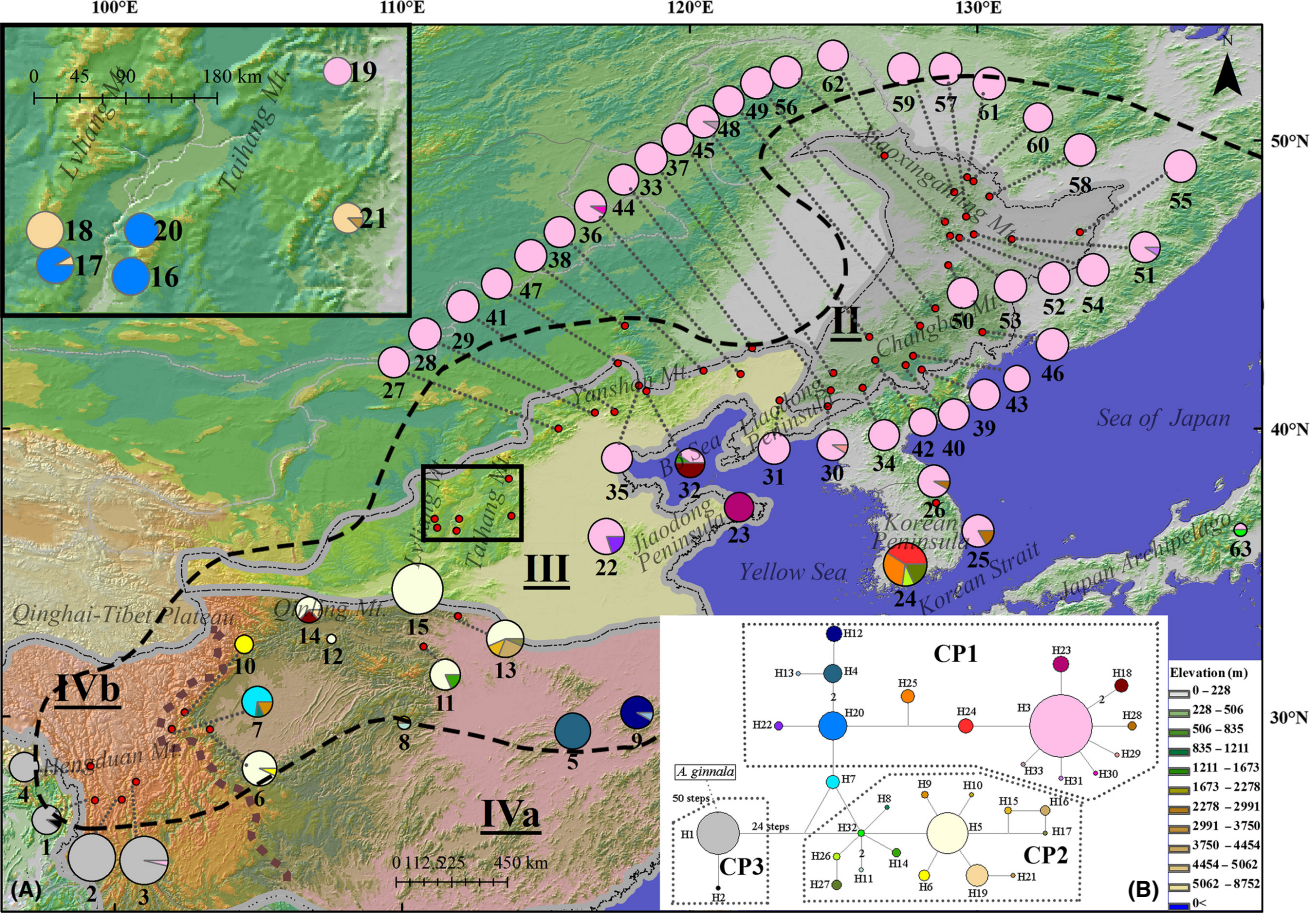
(A) Geographic distribution of the 33 chloroplast haplotypes. The size of the pies corresponds to sample size of each population. The area in Taihang and Lvliang Mts.(in the black frame) is magnified at the top left. The distribution range of *Acer mono* represents by the black dash line was modified as Hsu (1992).The different vegetation regions were separated by gray borders and different shadow colors in the map. The brown dash line divided vegetation region IV (subtropical evergreen broad-leaved forest region) into two subregions. The borders of vegetation region and subregion were both drawn according to Zhang (2007). Vegetation region/subregion names were abbreviated as follows: II, temperate coniferous broad-leaved mixed forest region (gray shadow); III, warm temperate deciduous broad-leaved forest region (yellow shadow); IVa, eastern subtropical evergreen broad-leaved forest region (red shadow); IVb, western subtropical evergreen broad-leaved forest region (red shadow). (B) The median-joining network of 33 cpDNA haplotypes. The size of pies is proportional to the haplotype frequency. Solid lines represent mutational steps interconnecting two haplotypes. Each line represents 1 step, except for the lines with additional annotation. The three clades were indicated by surrounding gray dotted line according to consensus tree in Fig. 4.

We sampled leaves of 1236 individuals from 63 populations of *A. mono* throughout its distribution range (Table S1; Fig. 2A). Sampled trees were at least 30 m apart to minimize collection of closely related individuals. Samples were desiccated in silica gel. Genomic DNA was extracted using a plant genomic kit (Tiangen, Beijing, China). Sampled populations were in the three vegetation regions of China, Japan, and South Korea. A total of 25 populations were located in region II, 20 populations in region III, 10 populations in region IVa, four populations located in the region IVb, and three populations in Korea. One population was located in Japan (Table S1).

### CpDNA sequencing

Two intergenic spacers within the chloroplast genome, *psbA*-*trnH* and *trnL*-*trnF*, and an intron of *rpl16* (Table S2) were amplified and sequenced for the samples (*N*_*C*_ = 731). PCR assays were performed in 40 *μ*L containing 10–20 ng of template DNA, 1× buffer, 10 mmol/L Tris-HCl (*pH* = 8.3), 50 mmol/L KCl, 200 mmol/L each dNTP, 2.0 mmol/L MgCl_2_, 0.1 mmol/L each primer, and two units of Taq (TaKaRa, Dalian, China). PCR amplifications were performed as follows: an initial denaturation step at 94°C for 5 min, followed by 24 cycles of 50 sec at 94°C, 50 sec at the annealing temperature (Tables S2) and 50 sec at 72°C, with a final extension step at 72°C for 10 min. PCR products were sequenced at the Beijing branch of the Beijing Genomics Institute. All the cpDNA sequences were uploaded to the GenBank. The GenBank accession numbers of these sequences were KC767550–KC767586.

### Nuclear microsatellite genotyping

Six nuclear microsatellite markers developed for *A*. *mono*(Kikuchi and Shibata 2008) and *A*. *yanbiense*(Zhao et al. 2011) were used to determine the nuclear genotypes of all samples (*N*_*M*_ = 1236, Table S1). The PCR conditions were the same as for the amplification of the chloroplast intergenic fragments (see above), except for the volume (20 *μ*L) and the annealing temperature (Table 1). The PCR products were genotyped using a 3730XL automated Genetic Analyser (Applied Biosystems, Foster City, CA). Allele sizes were determined in GENEMAPPER (version 3.7, Applied Biosystems).

**Table 1:** Details of 6 nuclear microsatellite loci

Locus	AM116	AM118	AM340	AM607	AY05	AY14	Average
*A*	38	16	36	25	31	20	27.6667
*H*_*t*_	0.9270	0.8410	0.8770	0.9050	0.9010	0.7460	0.8860
*H*_*o*_	0.8340	0.676**	0.7850	0.795***	0.776***	0.627*	0.749***
*H*_*e*_	0.8310	0.6940	0.7830	0.8330	0.8250	0.6550	0.7700
*F*_*ST*_	0.096***	0.170***	0.102***	0.111***	0.075***	0.113***	0.111***
*G'*_*ST*_	0.662***	0.602***	0.503***	0.681***	0.539***	0.363***	0.540***
Annealing temperature(°C)	57	54	57	62	50	58	
*P* (HWtest(*F*_*is*_))	0.6470	0.0010	0.4366	0.0000	0.0000	0.0124	0.0000

Number of alleles (*A*), total genetic diversity over all populations (*H*_*T*_), expected heterozygosity (*H*_*E*_), observed heterozygosity (Ho), *P* value of Hardy–Weinberg equilibrium test (*P*(HWtest(*F*_*is*_))) are shown for each loci.

*** *P* < 0.001,

** *P* < 0.005,

* *P* < 0.01.

### Chloroplast DNA sequence analysis

#### Sequence characteristics, phylogenetic analysis, and SAMOVA

Chloroplast sequence data were edited and aligned using Codoncode Aligner3.6.1 (http://www.codoncode.com/aligner/) with the Clustal module. The genetic diversity of populations and vegetation regions were calculated in Arlequin 3.5 (Excoffier and Lischer 2010). The diversities determined for vegetation regions were based on all the analyzed individual data per region. Haplotypes were determined in DNAsp 5.10.01 (Rozas et al. 2003) including the gaps. A median-joining haplotype network was constructed in NETWORK 4.6.1.1 (Bandelt et al. 1999), with deletions randomly coded as new bases. The composition of haplotypes in each population was mapped in Arcmap 9.3 (ESRI. Inc, RedLands City, CA, USA). To yield a more credible phylogenetic relationship, both maximum-likelihood and maximum-parsimony method in Mega5 (Tamura et al. 2011) were utilized in the inference. Branch support was tested via bootstrap analysis with 1000 replications.

The genetic structure and the potential genetic barriers for populations were analyzed using a spatial analysis of molecular variance (SAMOVA; Dupanloup et al. 2002). For each user-defined group number (*K*), the program simulated population groups with the highest variance among groups (*F*_*CT*_). We calculated the *F*_*CT*_ value for each group number from 2 to 13 and set the number of simulated annealing processes to 100.

### Divergence time

**Figure 3:**
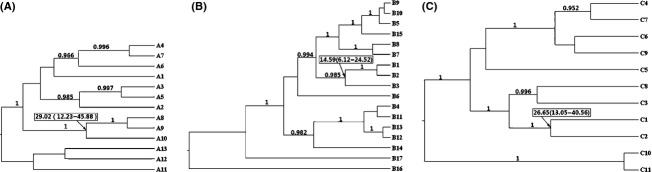
Phylogenetic trees based on cpDNA haplotypes of *Acer mono* and other species in Genus *Acer*. (A) Tree for *psbA*-*trnH*; (B) tree for *trnF*-*trnL*; (C) tree for *rpl16*. Values in boxes are the coalescence times of the 3 *A*. *mono* cpDNA haplotypes and their 95% confidence intervals. Values on the branches are the posterior probabilities of the branches. Detailed information on all of the haplotypes is provided in Table S4.

To estimate the divergence time of the major species lineages, we used a fossil age of 65 Mya as the calibration point, which has been reported as the earliest divergence time between *Acer* and *Dipteronia* (Renner et al. 2007). We also sequenced five additional *Acer* species (a total of 44 individuals) belonging to four sections of the genus (Table S3). The GenBank accession numbers of the sequences of additional species were KC767587–KC767638. We downloaded the same three chloroplast fragments from GenBank for both *Dipteronia* species (Table S3). For *A mono*, we only chose the three most common haplotypes (H1, H3, and H5; Fig. 2A, Table S3, Fig. 3) as representatives. We applied a Bayesian inference process in Beast 1.7.4 (Drummond and Rambaut 2007) independently for each chloroplast fragment using a Yule speciation process tree model and an uncorrelated lognormal clock model. Inference was conducted with an MCMC chain length of 10^7^ and 10^4^ recorded trees. The goodness of the inference was checked by evaluating the ESS value in Tracer 1.5 (Rambaut and Drummond 2009b), and the tree topology and crown age of *A. mono* trees were visualized in FIGTREE v1.3.1 (Rambaut and Drummond 2009a).

We applied a similar BEAST analysis to all *A. mono* haplotypes. We used the same settings as in the first step, with the exception of a constant-size coalescent tree prior and the timing of the MRCA (most recent coalescent ancestral) of *A. mono* clade as a normal distribution with mean (±1 SD) = 23.42 (±5) Mya (see Results, Fig. 3). Two *Acer* species (*A*. *ginnala* and *A*. *caudatifolium*, Table S3) were treated as an outgroup.

### Microsatellite data analysis

#### Genetic diversity and differentiation at each locus

For each microsatellite locus, the number of alleles (*A*), total genetic diversity over all populations (*H*_*t*_), expected heterozygosity (*H*_*e*_) and observed heterozygosity (*H*_*o*_) were calculated using FSTAT 2.9.3 (Goudet 2001). The genetic differentiation indices *F*_*ST*_ (Wright 1949) and *G'*_*ST*_ (Nei 1973) were evaluated for each locus in MSA 4.05 (Dieringer and Schlötterer 2003) with 10,000 permutations. Hardy–Weinberg tests were conducted in Genepop v4.2 (Rousset 2008) using default settings.

### Population genetic diversity and identification of genetic barriers

For each vegetation region and all populations, excluding populations 8, 10, 12, and 63 which all consisted of less than five individuals, genetic diversities were evaluated based on the number of effective alleles (*AR*), private allele frequency (*PAR*), expected heterozygosity (*H*_*E*_), and observed heterozygosity (*H*_*O*_) using GenAIex v6.1 (Peakall and Smouse 2006). Conformance tests to Hardy–Weinberg equilibrium were analyzed in Genepop v4.2.

Potential genetic barriers indicated by nuclear microsatellites were identified using Monmonier's maximum difference algorithm applied in Barrier v2.2 (Manni et al. 2004). The input Nei's *Da* genetic distance matrix (Nei and Chesser 1983) was calculated in MSA 4.05, with an additional 999 matrices generated through bootstrap analysis.

### STRUCTURE analysis

The model-based Bayesian algorithm in STRUCTURE 2.3.4 (Falush et al. 2007) detected population structure within *A. mono* populations. STRUCTURE analysis was run for 200 000 MCMC cycles following 100 000 burn-in cycles, using an admixture model with correlated allele frequencies. Ten independent runs were performed for each *K* value from 1 to 20. The trends of the log-likelihood value of the data (*lnP*(*D*)) and ΔK, introduced by Evanno et al. (2005), were used together to determine the number of potential gene pools.

A neighbor-joining tree was constructed in Mega 5 to reflect the genetic relationship of the defined gene pools.

### The Ecological Niche Model (ENM)

ENM for predicting the potential distribution of *A. mono* was constructed by relating modern distribution records and bioclimatic variables using the maximum entropy modeling technique (Maxent; Phillips et al. 2006). Modern distribution records were obtained from the Chinese Virtual Herbarium (http://www.cvh.org.cn/) as well as the 63 records from this study. In total, 261 presence records of *A. mono* were obtained from throughout the species range. Six bioclimatic variables (Hijmans and Graham 2006) at a 2.5-arcmin resolution were used to model the species' niche: (1) temperature seasonality; (2) mean temperature of the warmest quarter; (3) mean temperature of the coldest quarter; (4) annual precipitation; (5) precipitation of the wettest quarter; and (6) precipitation of the driest quarter. These six variables are common in ENM modeling of plant species (Sakaguchi et al. 2012; Chen et al. 2012; Qi et al. 2012) and play critical roles in species distribution (Woodward and Williams 1987). Avoidance of highly correlated variables helps prevent overfitting as well as inaccurate distribution prediction (Peterson and Nakazawa 2008). To test the performance of each model, 25% of the data in each run was randomly selected by Maxent and compared with the model output generated with the remaining data. Model validation was performed using default settings, with 10 replications. The area under the ROC curve (AUC) was calculated for each run, which indicates model accuracy (Fawcett 2006).

The established model was projected onto the climatic conditions reconstructed during the LGM, as simulated by MIROC3.2, provided by the Palaeoclimate Modelling Intercomparison Project (http://pmip2.lsce.ipsl.fr/). We utilized seafloor topography data (ETOPO1) produced by the National Geophysical Data Center of the National Oceanic and Atmospheric Administration (Boulder, CO) to estimate the paleocoast lines (−130 m compared with the present) and the paleoclimate surfaces of the exposed seafloor area during the LGM. The LGM paleoclimate layers were at a 2.5-arcmin resolution.

## Results

### Chloroplast sequences

#### Chloroplast haplotype variation, network, and distribution

The total alignment length of the three chloroplast fragments was 2140 bp, which had 33 haplotypes with 44 substitutions and 10 indels (Table S4). Most populations had only 1 haplotype, but populations 13 and 24 had 4 haplotypes. There was an average of 1.4 haplotypes per population and the nucleotide diversity (*π*) was 3.49 × 10^−3^ (Table 2).

**Table 2:** Genetic diversity results of three lineages of cpDNA

	CP1-NE	CP2-SE	CP3-SW	Overall
*N*	542	117	72	732
*N*_*h*_	15	15	2	33
*H*	0.339	0.639	0.028	0.630
*π* (10^−3^)	0.5161	0.6304	0.01349	3.491

Number of individuals (*N*), number of haplotypes (*N*_*h*_), haplotype diversity (*H*); nucleotide diversity (*π*).

**Figure 4:**
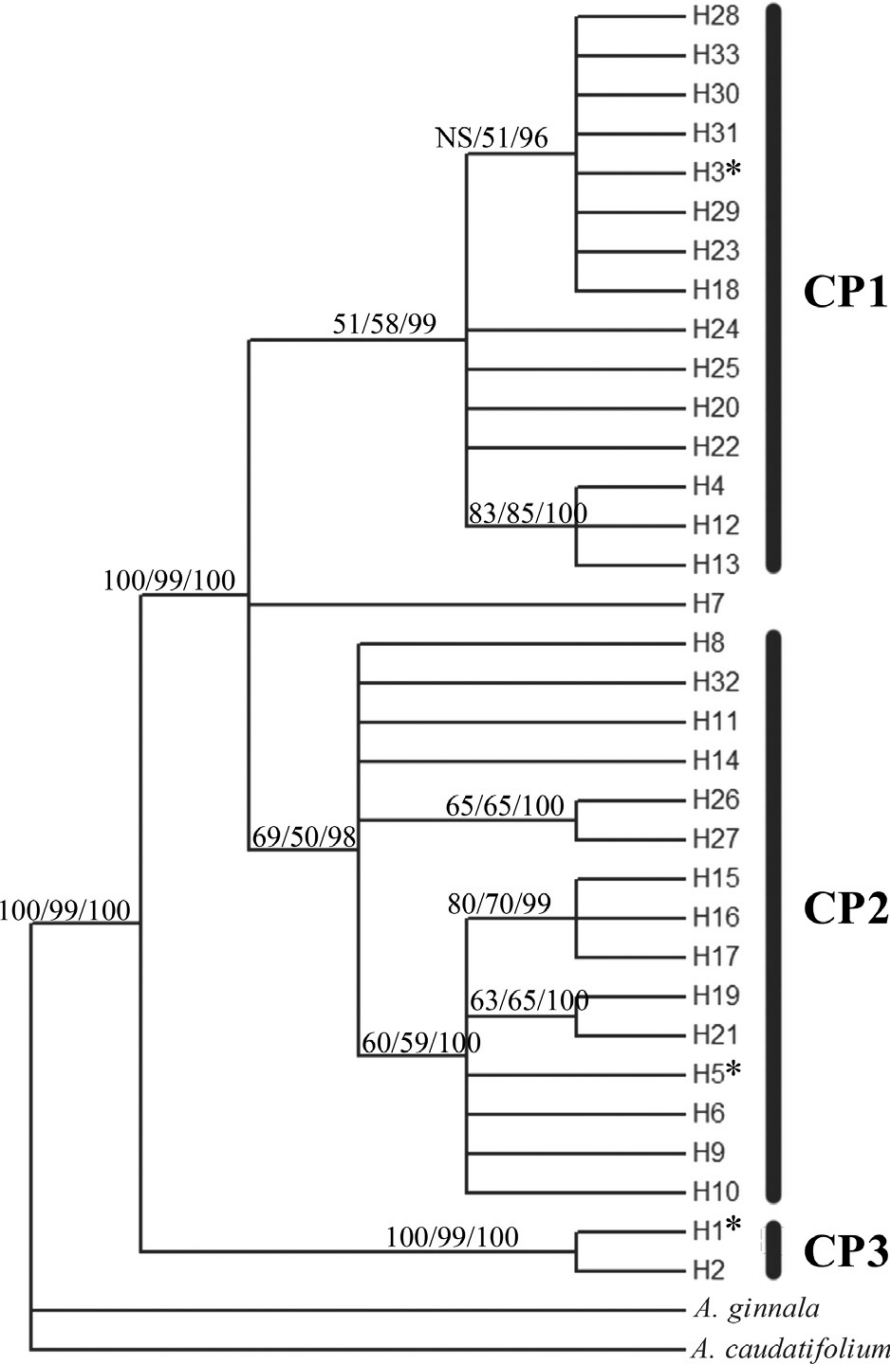
Rooted consensus trees for the chloroplast haplotypes. The values on the branches are bootstrap support values for the maximum-likelihood (left) and maximum-parsimony (middle) analyses and the posterior probability (right) of the Bayesian analysis. The sequences of haplotypes with “*”were used in construction of the phylogenetic trees in Fig. 3.

In the rooted phylogenetic consensus tree (Fig. 4), these haplotypes formed two well-supported clades separated by 25 mutation steps (Fig. 2B). The first clade (CP3) contained only H1 and H2, which occurred in subregion IVb (southwest China). The remaining 31 haplotypes constituted the second clade, which was subdivided into 1 basal haplotype (H7) and two subclades: CP1 and CP2 (Figs. 2B, 4). CP1 was mainly found from the north edge of region III and region II (central China to northeast China), where H3 was the predominant haplotype. CP2 was largely restricted to region IVa (southeast China), with H5 and H19 dominating in this region. H7, a basal haplotype of subclades CP1 and CP2, was located in the boundary of region IVa and IVb (population 7). The haplotype groups identified in the parsimony haplotype network (Fig. 2B) were consistent with the MP tree clades of CP1-CP3 (Fig. 4). Among the three lineages, CP2 had the greatest genetic diversity (*H* = 0.639, *π* = 0.6304 × 10^−3^), while CP3 had the lowest genetic diversity (*H* = 0.028, *π* = 0.01349 × 10^−3^). The diversity of CP1 was intermediate (*H* = 0.339, *π* = 0.1561 × 10^−3^) (Table 2).

### Molecular dating

The estimated crown age of *A. mono* was 23.42 Mya (95% HPD:15.29–32.85), suggesting a Miocene split between the two clades. In contrast, the CP2 and CP1 subclades likely diverged in the late Pliocene (6.98 Mya, 95% HPD: 4.127–9.841). The time of the most recent common ancestor for CP1 was estimated to be 4.127 Mya (95% HPD: 2.222–6.349), while that for CP2 was 4.127 Mya (95% HPD: 2.222–6.032), and that for CP3 was 0.952 Mya (95% HPD: 0.000–2.857). Based on the cpDNA chronogram, BEAST analysis provided an average substitution rate of 3.15 × 10^−10^ s/s/y, ranging from 2.89 × 10^−10^s/s/y (*rpl16*) to 3.50 × 10^−10^s/s/y (*psbA*-*trnH*).

### Nuclear microsatellite diversity and population structure

For the six microsatellite loci, there was a mean of 27.7 alleles per locus (range 16 to 38). The total genetic diversity over all populations, *H*_*t*_, was 0.886 (0.746–0.926), and the genetic differentiation was significant for all loci (*F*_*ST*_ = 0.111, *G'*_*ST*_ = 0.540, *P* < 0.001, Table 1). The mean (±SD) number of effective alleles for all populations was 5.339 (±1.295), and the expected mean heterozygosity was 0.760 (±0.067). A total of 11 populations showed significant departure from Hardy–Weinberg equilibrium (*P* < 0.05, Table S1).

**Figure 5:**
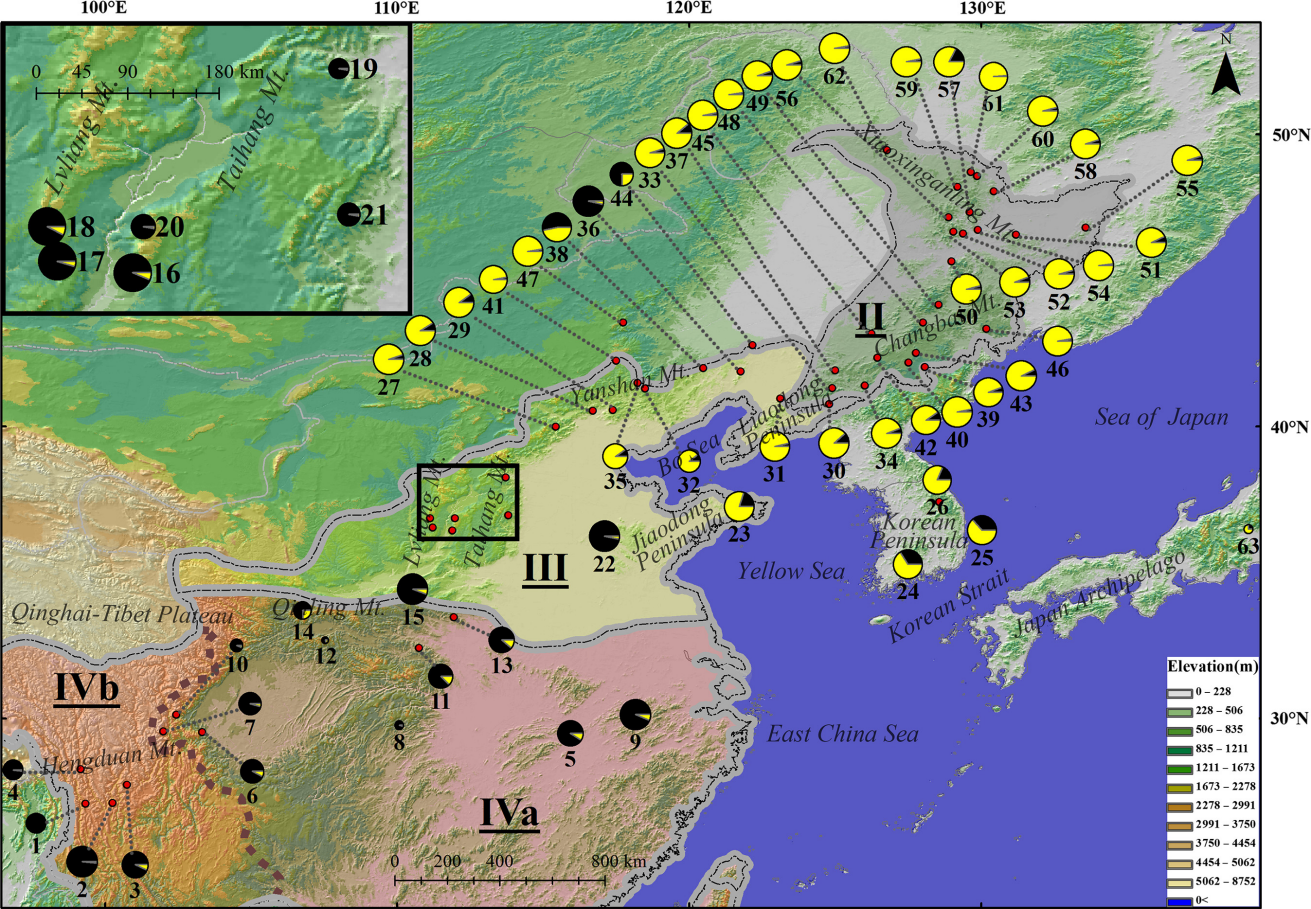
Geographic distribution of gene pools (*K* = 2). The size of the pies corresponds to sample size of each population. The area in which the Taihang and Lvliang Mts. are found is magnified at the top left. The different vegetation regions were separated by gray borders and different shadow colors in the map. The brown dash line divided vegetation region IV (subtropical evergreen broad-leaved forest region) into two subregions. The borders of vegetation region and subregion were both drawn according to Zhang (2007). Vegetation region/subregion names were abbreviated as followed: II, temperate coniferous broad-leaved mixed forest region (gray shadow); III, warm temperate deciduous broad-leaved forest region (yellow shadow); IVa, eastern subtropical evergreen broad-leaved forest region (red shadow); IVb, western subtropical evergreen broad-leaved forest region (red shadow).

**Figure 6:**
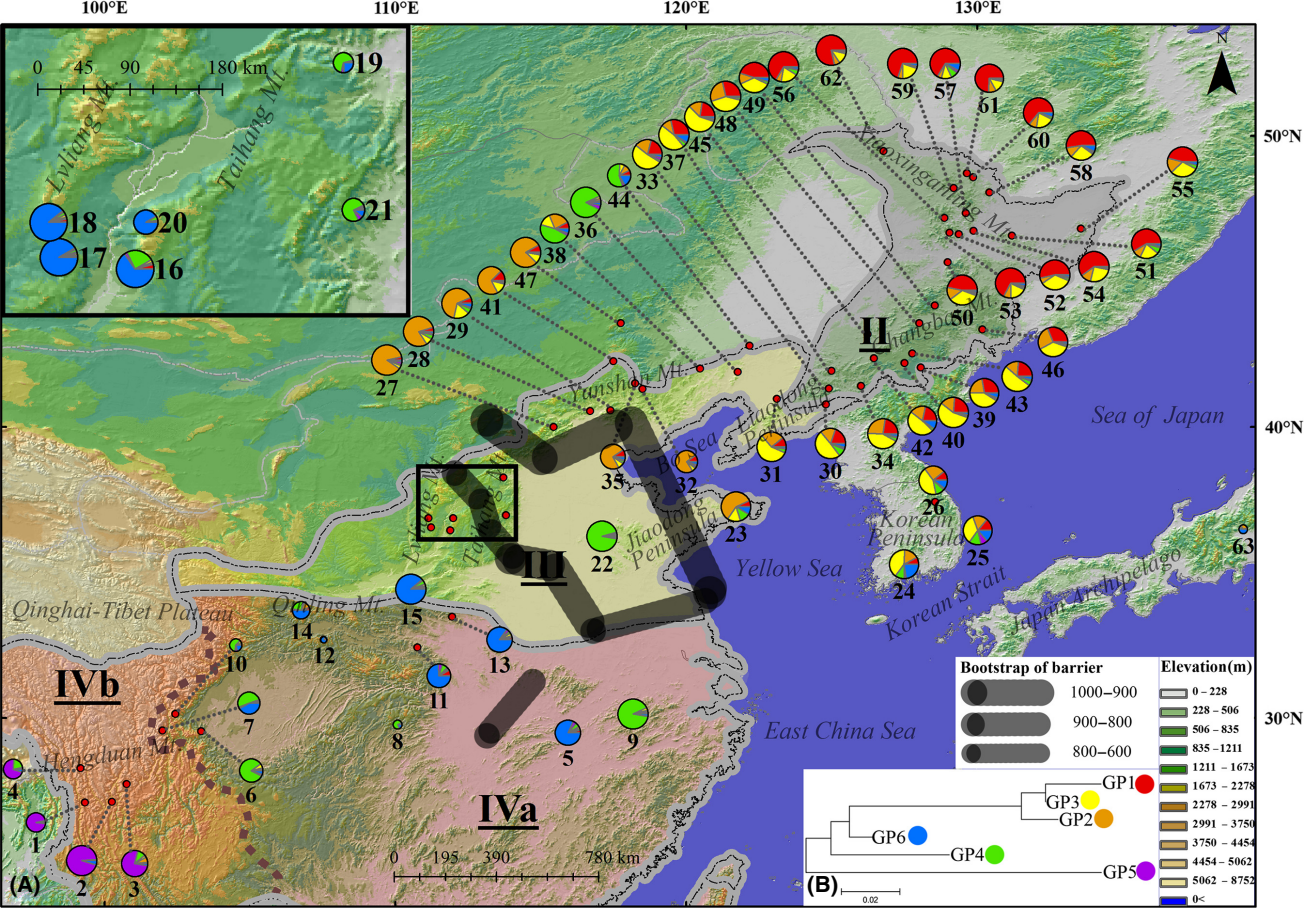
(A) Geographic distribution of gene pools (*K* = 6) and genetic barriers. The size of the pies corresponds to the sample size of each population. Black lines are genetic barriers, with the width representing the bootstrap value of the barrier. The area in which the Taihang and Lvliang Mts. are found is magnified at the top left. The different vegetation regions were separated by gray borders and different shadow colors in the map. The brown dash line divided vegetation region IV (subtropical evergreen broad-leaved forest region) into two subregions. The borders of vegetation region and subregion were both drawn according to Zhang (2007). Vegetation region/subregion names were abbreviated as followed: II, temperate coniferous broad-leaved mixed forest region(gray shadow); III, warm temperate deciduous broad-leaved forest region (yellow shadow); IVa, eastern subtropical evergreen broad-leaved forest region (red shadow); IVb, western subtropical evergreen broad-leaved forest region (red shadow). (B) Neighbor-joining tree for six gene pools.

In the STRUCTURE analysis, *ΔK* was highest for *K* = 2 (387.4), but the log-likelihood value of the data (*lnP*(*D*)) was the highest for *K* = 6 (Fig. S1). The two groups assigned by the *K* = 2 scenario were further divided into three groups, respectively, in the *K* = 6 scenario (Figs. 5, 6). Both the *K* = 2 and *K* = 6 scenarios are presented here. For *K* = 2, the boundary of two gene pools (the north group and the south group) was detected predominantly at the central area of Taihang Mt. (Fig. 5). For *K* = 6, the northern group was divided into GP1 (Xiaoxingan Mt.), GP2 (Yanshan Mt.), and GP3 (Korean Peninsula and south of Changbai Mt.), while the southern group was divided into GP4 (subtropical China), GP5 (Hengduan Mt.), and GP6 (north of Qinling Mt.).

Two genetic barriers with high bootstrap support were detected. One was located between north of Taihang Mt. and Yanshan Mt., extending south to the Jiaodong Peninsula. The other barrier was on border of the south of Taihang Mts. and Qinling Mt., extending north to the valley between Taihang and Lvliang Mt. (Fig. 6A).

### Ecological niche model

**Figure 7:**
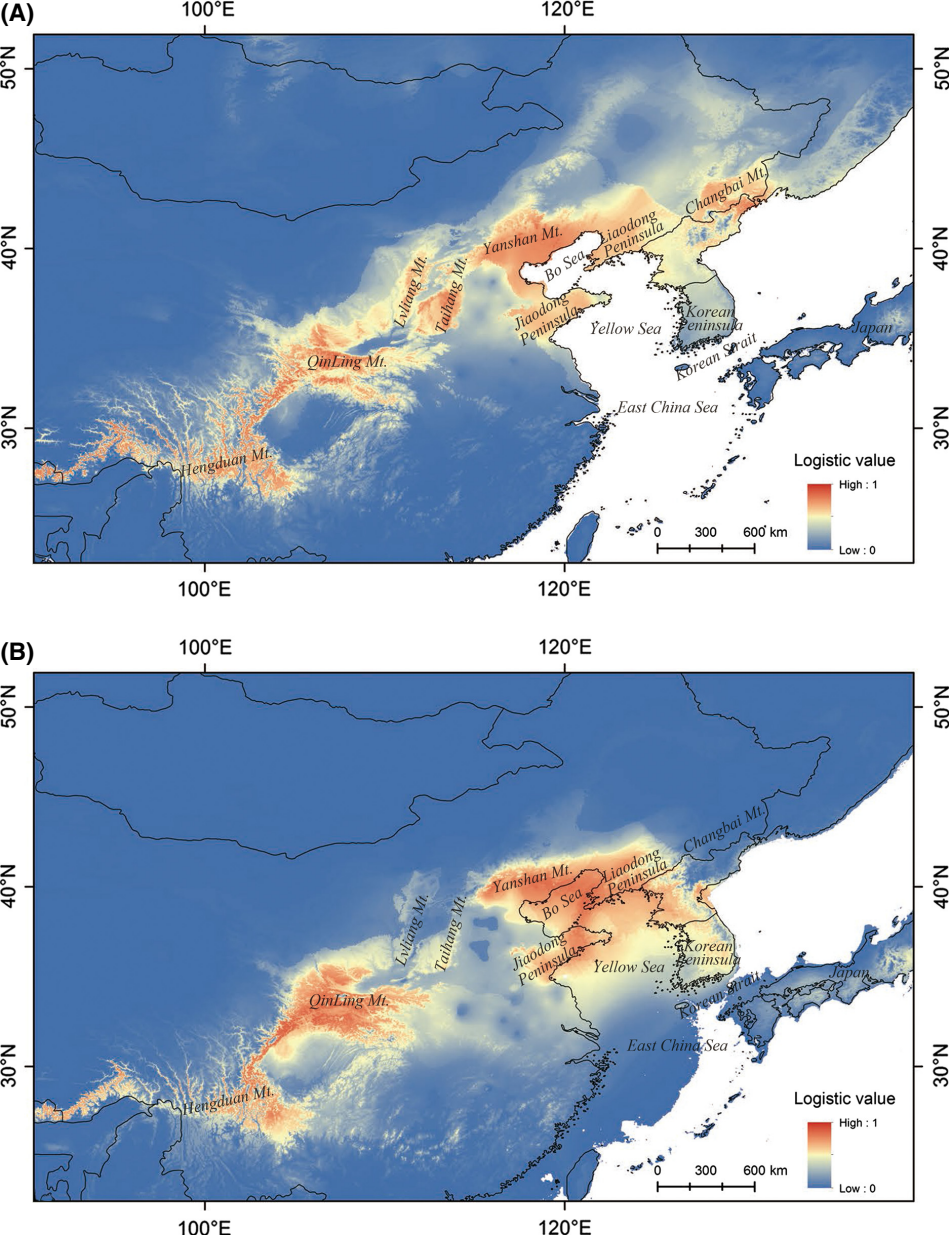
(A) The current species distribution of *Acer mono* simulated using a niche model. (B) The species distribution of *A. mono* simulated during the LGM using a niche model.

The Maxent model had high predictive power [AUC = 0.934 ± 0.011 (mean ± SD)]. Under the present climate, five areas of *A. mono* distribution were predicted with a high probability: (1) middle and high elevation areas of Hengduan Mt.(region IVb); (2) Qinling Mt., at the boundary between region III and IV; (3) Taihang and Lvliang Mts. in the southwest region III; (4) Bo Sea Coastal region in northeast region III, including Yanshan and Qianshan Mts. and the Jiaodong Peninsula; and (5) Changbai Mt. in region II and northeast North Korea. The predicted distribution based on the model was similar to the actual species distribution (Figs. 2A, 7A).

Under the LGM climate, the species distribution in south China appeared little changed. In contrast, extremely low distribution probabilities were predicted for north of Changbai Mt. (region II) during the LGM. Additionally, the distribution probabilities in the Taihang-Lvliang Mts. Range decreased, while the distribution area in the Bo Sea Coastal region increased (Fig. 7B).

## Discussion

### Retreat and expansion of populations in Region II

Both the markers of the chloroplast genome and the nuclear genome revealed a genetic diversity gradient across latitude in *A. mono* populations. Genetic diversity and privacy decreased from the south to the north; populations in region II had the lowest diversity and privacy (Table S1). In region II, the chloroplast markers showed little genetic structure and only one chloroplast haplotype (H3) dominated in all populations. The nuclear microsatellite markers revealed more genetic structure (GP1, GP2, and GP3), but region II results still indicated a single origin, with limited private sites (Fig. 6B). The ENM results provided a historical perspective for genetic data interpretation. During the LGM, suitable habitat for *A. mono* was rare in region II (Fig. 7B). Reduced temperature and humidity during the LGM were the most critical factors. The modeling results were in accord with pollen records, which indicated that Changbai Mt. and the areas north were dominated by either cold temperate coniferous forests or forest steppes and the development of modern mixed forests did not begin until 13,000 years ago (Liu et al. 2008; Xia et al. 1983).

The genetic data and the ENM simulation indicated that during the LGM, *A. mono* retreated to region III, which was farther north than previously suggested (Harrison et al. 2001). Both chloroplast and nuclear fragments data showed distinct genetic differences between populations in region IV and that in the northern regions III and II. This genetic variation occurred and expanded much earlier than the LGM. The coalescence time of the chloroplast lineage (CP1) can be traced back to Pliocene (Neogene), 4.127 Mya (2.222–6.349), and even the coalescence time of haplotype H3 and its closely related haplotypes (mainly found in regions II and III) can be traced back to the early Pleistocene, 1.587 Mya (0.635–2.540). Additional evidence comes from the ENM results. During the LGM, northern region III probably had a large area of habitat suitable for *A. mono* encompassing the northern edge of region III (Yanshan Mt. and Liaodong Peninsula) to Jiaodong Peninsula and the Korean Peninsula. This northern region III area might represent an independent distribution center during the glaciation, which was different from another center in Qinling Mt.. This hypothesis is supported by the high genetic diversity and privacy in the northern region III. The genetic composition of region II was nested within northern region III, suggesting that region II populations probably originated from expansion of this northern distribution center after the LGM.

The different genetic structure between chloroplast and microsatellites in northern region III, region II, and the Korean Peninsula might originate from difference in heredity over time and mutation rate. A lower mutation rate and wind-mediated seed dispersal resulted a homogeneous genetic structure in the chloroplast makers, while a higher mutation rate combined with relatively restricted dispersal related to bee and fly pollination in the nuclear genome led to a more structured pattern. Based on nuclear microsatellites, a detailed migration route for *A. mono* can be suggested. The northern nuclear genetic group can be further divided into three gene pools in the STRUCTURE analysis (Fig. 7B). Region II was dominated with GP1 and GP3, which were rare in Yanshan Mt., which was mainly GP2. This nuclear pattern suggested that the populations in region II probably recovered from south of Changbai Mt. or Korea Peninsula.

*Acer mono* was not unique in its survival during the LGM in northern region III. Two other dominant canopy species, *J. mandshurica* and *Q. mongolica*, have a similar history of population demographics: their populations retreated to south of Changbai Mt., with a relatively high genetic diversity remaining in northern region III (Zeng et al. 2011; Bai et al. 2010). For *Pinus koraiensis*, a constructive forest species in region II, diversity is low in region II, possibly as the result of a population bottleneck in the glacial period, with populations retreating south of Changbai Mt. (Aizawa et al. 2012). These tree species were canopy types that determined the forest structure in region II. Our study, in combination with previous work, presented a clear picture of vegetation history of the forests in region II, although greater clarity requires additional species of various taxonomic groups and life histories. Our study documented the important role of northern region III in the recolonization of modern species populations in region II.

### Consistency between intraspecific genetic diversity and species diversity pattern

Our genetic data demonstrated that the intraspecific distribution of genetic diversity in *A. mono* was consistent with the species diversity pattern on the SJFR mainland. At the species level, region IV (southern) had greater diversity and endemism than region II and III (northern); while within region IV, region IVb (western) was greater than region IVa (eastern). Hengduan Mt. in region IVb had the highest total number of seed plants species (7954) and endemic species (2988), while region III (north China) and region II (northeast China) had the lowest species diversity (3358 and 1776 for region III and II, respectively) and endemism (192 and 124 for region III and II respectively; Ying 2001; Wang et al. 1997). For *A. mono*, we observed a similar genetic diversity pattern. Region IV had the greatest number of chloroplast haplotypes (*H* = 19) and privacy (*Private sites* = 37). Diversity decreased with increasing latitude, and region II had the lowest diversity in both abundance and endemism (*H* = 4, *Private sites* = 2). Microsatellite data revealed a pattern similar to that of the chloroplasts (Table S1), yet discrepancies existed from the species level pattern.

A major discrepancy was located in region III, which contains a very similar high nuclear microsatellite diversity (*H*_*E*_ = 0.870, *AR* = 5.02; in region IV: *H*_*E*_ = 0.877, *AR* = 5.12), but low privacy (*PAR* = 1.59; in region IV: *PAR* = 2.18). This genetic diversity pattern may have originated from the transition status of region III between region II (northern) and region IV (southern). Region III held both chloroplast haplotypes from CP1 and CP2, and nuclear gene pool of both southern and northern ancestral origin (GP2, GP4, GP6). There is a clear boundary between southern and northern genetic types located in the north of Taihang Mt. (Fig. 6A), as well as populations located within the same latitude in the east. Although few populations gave evidence for hybridization or coexistence between southern and northern genetic types, this transition status within the same region seemed to have increased genetic diversity in abundance, but not privacy. A transition zone or hybridization area between southern and northern genetic types is not unique to *A. mono*. For example, the species complex *Q*. *mongolica* and *Q*. *liaotungensis* formed a genetic crossing zone on north Taihang Mt. and Yanshan Mt. (Zeng et al. 2010, 2011). The cpDNA haplotypes of another important northern tree species, *J*. *mandshurica*, also indicted a genetic crossing zone on north Taihang Mt., as did that of its southern sister species, *J*. *cathyensis* (W.-N. Bai, unpubl. data). Similarly, the shrub *Ostryopsi davidiana* exhibited high genetic diversity on Taihang Mt., particularly in the northern region. Northern populations of *O. davidiana* shared haplotypes with southern populations (Tian et al. 2009). Region III has been characterized as a transition region at the species level for harboring species from both subtropical and temperate zones. Interestingly, the species composition in the north of Taihang Mt. (EW-trending) and Yanshan Mt. was significantly different from that in the south of Taihang Mt. (Liu et al. 2004; Ru and Zhang 1993, 2000; Wang et al. 2005). This reflected the correlation between intraspecific genetic diversity and species diversity patterns at local scales.

Our ENM results indicated that the transition status of *A. mono* in region III may reflect climatic oscillations. There were probably multiple isolated distribution centers for *A. mono* during the LGM. Low survival in the Taihang and Lvliang Mts. resulted in two separated major distribution regions during the LGM, one centered in the northern region III (Yanshan and the south of Changbai Mt.) and another in southern region III (Qinling Mt.) (Fig. 7B). These two ancient distribution centers appeared very limited mixture even nowadays, considering our extensive sample strategy in mainland of East Asia. The transition status, as well as persistent plant populations, allowed region III to significantly contribute to the preservation and intraspecific genetic diversity of temperate flora species. Physiographic heterogeneity may also contribute to this function of region III. The northwest area of region III is characterized by mountains, while the east consists of the Jiaodong and Liaodong Peninsulas, which are isolated hills. The eastern and western areas are connected by a vast plain. The diversity of this terrain favored the survival of plants within isolated refugia during periods of glaciation. This is in addition to the distribution centers in region III which contain the unique haplotypes of *A. mono* and many other temperate plant species (Bai et al. 2010; Zeng et al. 2010, 2011; Tian et al. 2009).

The Korean Peninsula, although surround by ocean, is similar to the environment of region III. During the glacial periods, the peninsula was connected with the mainland due to lower sea level (Fig. 7B). The chloroplast haplotypes were mainly northern types (H3, CP1) in the north of the peninsula, while they were a mixture of CP1 (northern) types and CP2 (southern) types in the southernmost population (id: 24, Fig. 2A). All four haplotypes were private in population 24. Nuclear microsatellite analysis revealed a prominence of northern genetic clusters but with a mixture in the southern populations (24 and 25) (Fig. 6A). These patterns suggested a transition role of the Korean Peninsula as region III.

Another discrepancy was observed between the two diversity levels located in Hengduan Mt. (subregion IVb), which harbored the most diverse and endemic species in East Asia and is one of the 25 worldwide biodiversity hotpots (Myers et al. 2000). For *A. mono*, however, the genetic diversity in subregion IVb was much lower than subregion IVa or region III in both chloroplast and microsatellite data, and approximately equal to region II (Table S1). This low diversity pattern may have resulted from the small sample size in subregion IVb. However, this region had extremely high privacy although only four populations were sampled. It had only two chloroplast haplotypes belonging to an independent and ancient lineage CP3 containing at least 25 mutation steps distant from haplotypes in other regions (Fig. 2B). It was the same in nuclear microsatellite data revealed a distinct gene pool GP5 in subregion IVb (Fig. 6A) with relatively high privacy (*PAR* = 1.66, Table S1). Another ancient haplotype H7 only occurred, at high proportion, in population seven which located in westernmost area of subregion IVa, close to IVb. The haplotype H7 was basal in the big lineage with CP1 and CP2. Collectively, the ancient genetic composition of subregion IVb and adjacent areas suggested the primordial status of Hengduan Mt. for *A. mono*, which was consistent with the hypothesis that Hengduan Mt. played as an origin and diversification center for genus *Acer* and family Aceraceae (Hsu 1983, 1998). This primordial status of this region appears to be true for many other temperate plant species, for example, *Spiraea japonica* (Zhang et al. 2006; Li et al. 2010), *Picea* (Li et al. 2010), *Taxus wallichiana* (Gao et al. 2007), *Dipentodon* (Yuan et al. 2008), and *Dysosma versipellis* (Qiu et al. 2006) and also for higher taxonomic categories (Li and Li 1993).

Physiographic complexity in subregion IVb played critical role in the formation and preservation of primitive biodiversity. First, Hengduan Mt. was an ancient land and began to uplift at the end of the Jurassic and it reached a 1000 m elevation during the early Tertiary (70–40 Mya) due to movement of the Himalayas (Li 1986). The mountain remained at approximately 1000 m until the early Pleistocene. The latest uplift of Hengduan Mt. did not begin until 2 Mya. Relatively stable topography over a long time interval provided excellent conditions for maintenance of genetic diversity and speciation. Second, the north-to-south valleys provided potential routes for species migration, while the steep mountains, with elevations exceeding 3000 m, may act as a barrier to dispersal between the east and west, especially for species with restricted dispersal ability (Zhang et al. 2011). Long-term isolation and genetic drift could lead to fixation of unique genetic compositions or speciation. Third, the dramatic altitude changes in this mountain range resulted in extremely diverse habitats from the tropical/subtropical regions in the valleys to arctic alpine regions. Adaptation to this range of conditions fostered considerable genetic divergence and speciation. All *A. mono* populations on Hengduan Mt. were found above 2000 m (Table S1). Although it was not clear whether adaptation has occurred in our maple trees, potential adaptation might promote divergence, which require further study in the future.

## Conclusion

We used chloroplast sequences and nuclear microsatellite markers to study a Tertiary relict temperate forest tree species *A. mono*. The results showed high genetic diversity throughout its range and a distinctive genetic structure. A continental ice cap did not cover the temperate deciduous broad-leaved and coniferous mixed forest region (II) in northeast China during the last glaciation maximum, but *A. mono* populations retreated to the south due to decreased temperature and humidity and formed a distribution center during LGM at the northern edge of the warm temperate deciduous broad-leaved forest region (III). Present day populations in region II probably recolonized from south of Changbai Mt. or from the Korean Peninsula.

In general, the intraspecific genetic diversity of the widespread species, *A. mono,* was consistent with the species level diversity pattern. Therefore, abundance and privacy were higher in the south than in the north. There were two exceptions. One was in region III where the populations with the highest genetic diversity probably originated from the transition between northern and southern genetic types. Another one was in the western subtropical evergreen broad-leaved forest subregion(IVb) with very low genetic diversity, probably due to the limited sample size in this region. Whereas the ancient lineage or haplotypes dominated in IVb and adjacent area (population 7) indicated the critical role of this area in species origin and diversification. By comparing the diversity pattern at two levels, we highlighted the significance of region III, including the Korean Peninsula, in reserving temperate floristic biodiversity during climatic oscillations and connecting the diversity between region IV (southern) and region II (northern). Physiographic heterogeneity represented by diverse terrain and landforms in region III may contribute to this vital function.

The Japanese Archipelago appeared to have closer chloroplast relationships with central China. However, there were insufficient samples from Japan to make further inference regarding the relationship between the Japanese Archipelago and the mainland.

## Supporting Information

**Figure S1.** *LnP*(*D*) and Δ*K* for each *K* in the STRUCTURE analysis.

**Table S1.** Details of sample localities for the 63 *Acer mono* populations studied. **Table S2.** The information of chloroplast DNA primers used in this study. Table S3. The information of haplotypes used to estimate the divergence time of *Acer mono*. **Table S4.** CpDNA haplotypes. **Table S5.** Results of analysis of molecular variance (AMOVA) of cpDNA haplotypes and nuclear microsatellite alleles frequencies for populations and population groups of *Acer mono*. **Table S6.** The SAMOVA of *Acer mono* based on cpDNA.
