# Supplementary figures and images for: Evolutionary history of a widespread tree species Acer mono in East Asia
Source: Ecol Evol. 2014 Oct 27;4(22):4332–45. doi: 10.1002/ece3.1278 (PMC4267871; doi:10.1002/ece3.1278)

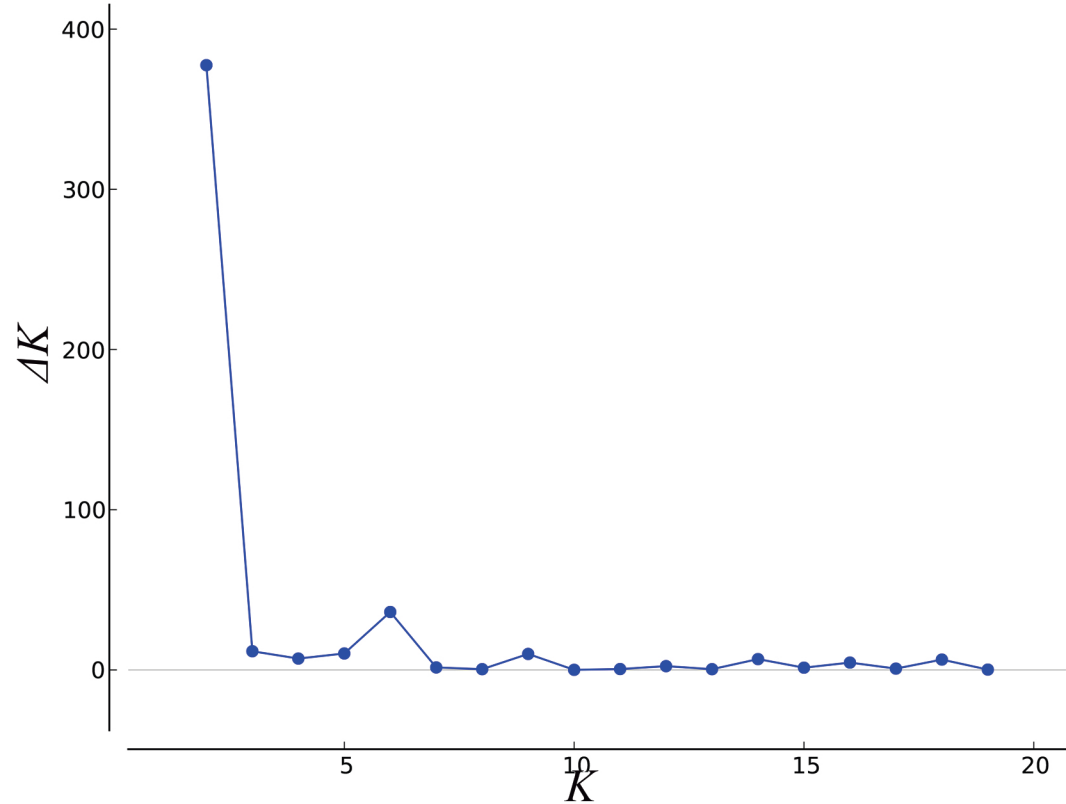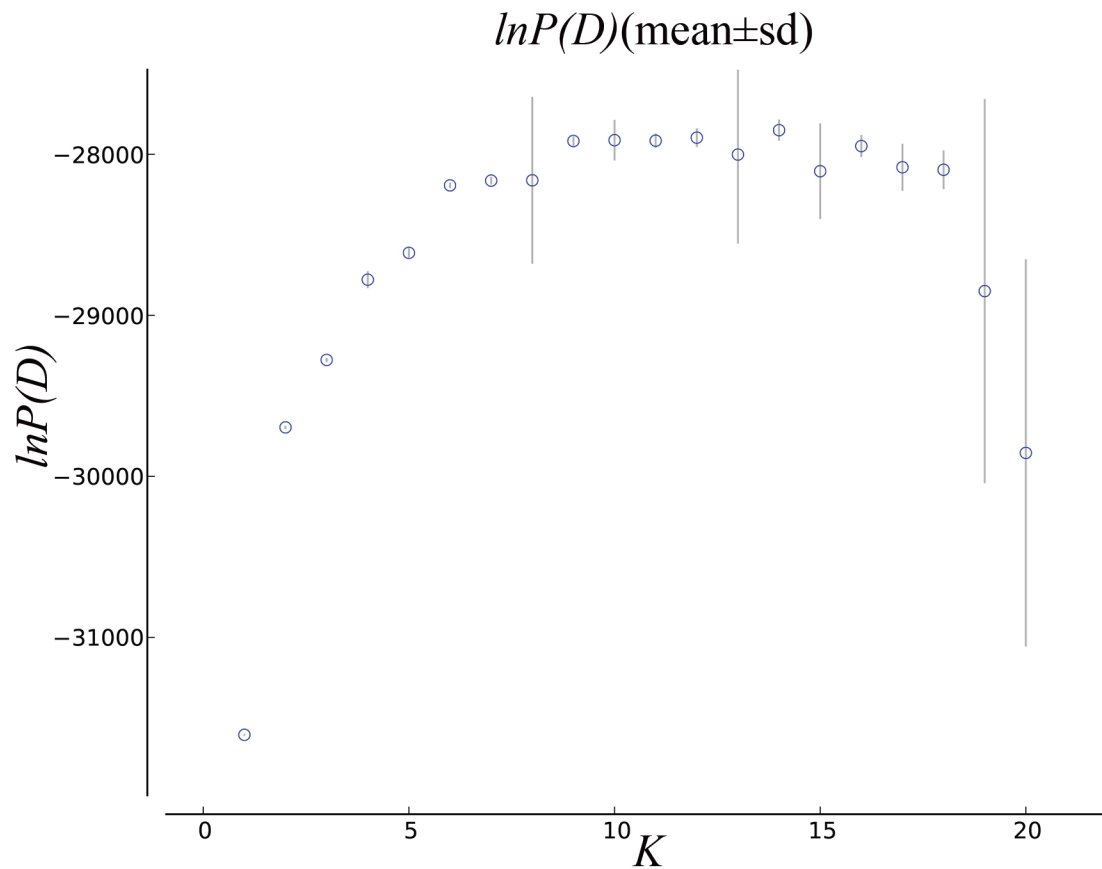

Supplement: Supplementary file 1 — Figure S1. LnP(D) and ΔK for each K in the STRUCTURE analysis. [file ece30004-4332-SD1.pdf]
