# Supplementary material for: Evolutionary history of a widespread tree species Acer mono in East Asia
Source: Ecol Evol. 2014 Oct 27;4(22):4332–45. doi: 10.1002/ece3.1278 (PMC4267871; doi:10.1002/ece3.1278)
Supplement: Supplementary file 2 [file ece30004-4332-SD2.doc]

| **Table S1 Details of sample localities for the 63 *Acer mono* populations studied.** | | | | | | | | | | | | | | |
| --- | --- | --- | --- | --- | --- | --- | --- | --- | --- | --- | --- | --- | --- | --- |
|  |  |  |  | **Nuclear Microsatellite** | | | | | | **Chloroplast DNA** | | | | |
| **Region/population** | **Longitude(°E)** | **Latitude(°N)** | **Alt (m)** | ***N_M_*** | ***AR*** | ***PAR*** | ***H_E_*** | ***H_O_*** | ***HW*** | ***N_C_*** | ***H*** | ***π*(10^-3^)** | **Haplotype** | **P****rivate sites** |
| **IV** |  |  |  |  | **5.12** | **2.18** | **0.877** | **0.695** |  |  | **19** | **6.6455** |  | **37** |
| **IVb** |  |  |  |  | **4.18** | **1.66** | **0.77** | **0.675** |  |  | **3** | **0.2678** |  | **24** |
| 1 Weixi | 99.32 | 27.10 | 2698 | 10 | 3.39 | 0.29 | 0.614 | 0.665 | 0.385 | 10 | 2 | 0.0489 | H1(9)H2(1) | 1 |
| 2 Shangri-La | 100.25 | 27.12 | 3048 | 24 | 3.76 | 0.13 | 0.700 | 0.733 | 0.238 | 24 | 1 | 0.0000 | H1(26) | 0 |
| 3 Lugu Lake | 100.75 | 27.74 | 2902 | 22 | 3.60 | 0.05 | 0.694 | 0.602 | 0.002* | 27 | 2 | 0.5249 | H1(26)H3(1) | 23 |
| 4 Baima Mt. | 99.17 | 28.29 | 3034 | 10 | 4.09 | 0.13 | 0.722 | 0.696 | 0.128 | 10 | 1 | 0.0000 | H1(10) | 0 |
| **IVa** |  |  |  |  | **5.00** | **1.52** | **0.863** | **0.709** |  |  | **16** | **1.5020** |  | **12** |
| 5 Lu Mt. | 115.93 | 29.50 | 1107 | 17 | 8.84 | 0.31 | 0.568 | 0.574 | 0.249 | 15 | 1 | 0.0000 | H4(15) | 0 |
| 6 Emei Mt. | 103.32 | 29.55 | 1435 | 14 | 3.46 | 0.05 | 0.650 | 0.679 | 0.366 | 14 | 2 | 0.0699 | H5(13)H6(1) | 1 |
| 7 Hailuogou | 101.99 | 29.57 | 3061 | 13 | 4.20 | 0.03 | 0.706 | 0.640 | 0.010* | 11 | 3 | 0.4148 | H7(8)H8(1)H9(2) | 2 |
| 8 Tianping Mt. | 110.07 | 29.78 | 1026 | 2 | **--** | **--** | **--** | **--** | **--** | 2 | 2 | **--** | H10(1)H11(1) | 3 |
| 9 Huang Mt. | 118.16 | 30.14 | 1700 | 24 | 3.58 | 0.00 | 0.648 | 0.646 | 0.317 | 12 | 2 | 0.0814 | H12(11)H13(1) | 1 |
| 10 Labahe | 102.44 | 30.15 | 2214 | 4 | **--** | **--** | **--** | **--** | **--** | 4 | 1 | **--** | H6(4) | 0 |
| 11 Saiwudang | 110.75 | 32.43 | 1394 | 15 | 3.81 | 0.14 | 0.681 | 0.686 | 0.280 | 11 | 2 | 0.1601 | H5(9)H14(2) | 0 |
| 12 Micang Mt. | 107.53 | 32.69 | 1568 | 1 | **--** | **--** | **--** | **--** | **--** | 1 | 1 | **--** | H5(1) | 0 |
| 13 Baotianman | 111.95 | 33.49 | 1391 | 17 | 5.00 | 0.01 | 0.845 | 0.909 | 0.811 | 16 | 4 | 0.5436 | H5(9)H15(2)H16(4)H17(1) | 3 |
| 14 Wuliang Mt. | 106.76 | 33.71 | 1308 | 8 | 4.54 | 0.03 | 0.734 | 0.821 | 0.576 | 8 | 3 | 0.6399 | H5(5)H18(2)H19(1) | 0 |
| **III** |  |  |  |  | **5.02** | **1.59** | **0.870** | **0.756** |  |  | **10** | **1.2334** |  | **4** |
| 15 Xiaoqinling | 110.53 | 34.43 | 1477 | 24 | 4.94 | 0.10 | 0.838 | 0.825 | 0.044* | 24 | 1 | 0.0000 | H5(30) | 0 |
| 16 Dananping | 111.89 | 36.46 | 1243 | 24 | 4.79 | 0.02 | 0.838 | 0.748 | 0.001* | 12 | 1 | 0.0000 | H20(12) | 0 |
| 17 Wulu Mt. | 111.22 | 36.56 | 1355 | 24 | 4.82 | 0.11 | 0.831 | 0.818 | 0.236 | 12 | 2 | 0.1630 | H19(1)H20(11) | 0 |
| 18 Yunmeng Mt. | 111.13 | 36.87 | 1422 | 24 | 4.89 | 0.09 | 0.845 | 0.861 | 0.491 | 12 | 1 | 0.0000 | H19(12) | 0 |
| 19 Tiangui Mt. | 113.71 | 38.26 | 922 | 7 | 12.49 | 0.02 | 0.660 | 0.730 | 0.815 | 7 | 1 | 0.0000 | H3(7) | 0 |
| 20 Mian Mt. | 111.98 | 36.87 | 1538 | 10 | 4.72 | 0.00 | 0.814 | 0.846 | 0.395 | 10 | 1 | 0.0000 | H20(10) | 0 |
| 21 Qingyazhai | 113.8 | 36.98 | 1309 | 8 | 4.67 | 0.09 | 0.785 | 0.845 | 0.849 | 8 | 2 | 0.0611 | H19(7)H21(1) | 1 |
| 22 Taishan | 117.1 | 36.25 | 770 | 24 | 11.58 | 0.03 | 0.586 | 0.610 | 0.405 | 15 | 2 | 0.3350 | H3(12)H22(3) | 1 |
| 23 Kunyu Mt. | 121.71 | 37.27 | 838 | 23 | 11.69 | 0.02 | 0.632 | 0.577 | 0.004* | 10 | 1 | 0.0000 | H23(10) | 0 |
| Number of sample size for nuclear microsatellite(*N_M_*,), number of effective alleles(*AR*); *PAR*, private allele frequency, expected heterozygosity(*H_E_*), observed heterozygosity(*H_o_*), *P* value of Hardy-Weinberg equilibrium test(*HW*), * means *P*<0.05, number of sample size for chloroplast DNA(*N_C_*); number of haplotypes(*H*) and nucleotide diversity(*π*) are shown for each population. | | | | | | | | | | | | | | |

| **Table S1 continuous** | | | | | | | | | | | | | | |
| --- | --- | --- | --- | --- | --- | --- | --- | --- | --- | --- | --- | --- | --- | --- |
|  |  |  |  | **Nuclear Microsatellite** | | | | | | **Chloroplast DNA** | | | | |
| **Region/population** | **Longitude(°E)** | **Latitude(°N)** | **Alt (m)** | ***N_M_*** | ***AR*** | ***PAR*** | ***H_E_*** | ***H_O_*** | ***HW*** | ***N_C_*** | ***H*** | ***π*(10^-3^)** | **Haplotype** | **Private sites** |
| 27 Dongling Mt. | 115.44 | 40.01 | 1431 | 24 | 3.75 | 0.05 | 0.724 | 0.663 | 0.007* | 11 | 1 | 0.0000 | H3(11) | 0 |
| 28 Yunmeeng Mt. | 116.70 | 40.55 | 1064 | 24 | 4.10 | 0.06 | 0.768 | 0.747 | 0.075 | 12 | 1 | 0.0000 | H3(12) | 0 |
| 29 Wuling Mt. | 117.39 | 40.58 | 1104 | 24 | 3.99 | 0.05 | 0.725 | 0.701 | 0.333 | 12 | 1 | 0.0000 | H3(12) | 0 |
| 31 Qian Mt. | 123.12 | 40.98 | 355 | 23 | 4.06 | 0.03 | 0.757 | 0.830 | 0.881 | 12 | 1 | 0.0000 | H3(12) | 0 |
| 32 Liaoheyuan | 118.50 | 41.31 | 1169 | 13 | 3.76 | 0.01 | 0.682 | 0.745 | 0.728 | 10 | 3 | 0.6227 | H3(4)H14(1)H18(5) | 0 |
| 35 Maojinba | 118.24 | 41.50 | 1114 | 16 | 4.20 | 0.01 | 0.761 | 0.759 | 0.339 | 11 | 1 | 0.0000 | H3(11) | 0 |
| 36 Daban | 121.77 | 41.90 | 452 | 24 | 4.34 | 0.09 | 0.738 | 0.749 | 0.488 | 12 | 2 | 0.0408 | H3(11)H30(1) | 1 |
| 38 Daheishan | 120.48 | 42.01 | 663 | 22 | 4.60 | 0.03 | 0.807 | 0.808 | 0.174 | 11 | 1 | 0.0000 | H3(11) | 0 |
| 41 Saihanba | 117.50 | 42.27 | 1370 | 20 | 3.57 | 0.00 | 0.681 | 0.694 | 0.078 | 11 | 1 | 0.0000 | H3(11) | 0 |
| 44 Daqinggou | 122.17 | 42.80 | 228 | 14 | 4.46 | 0.00 | 0.786 | 0.689 | 0.005* | 11 | 1 | 0.0000 | H3(11) | 0 |
| 47 Heshigten | 117.75 | 43.57 | 1199 | 24 | 3.42 | 0.03 | 0.644 | 0.607 | 0.196 | 12 | 1 | 0.0000 | H3(12) | 0 |
| **II** |  |  |  |  | **4.31** | **1.07** | **0.785** | **0.752** |  |  | **4** | **0.0077** |  | **2** |
| 30 Baishi | 124.80 | 40.78 | 783 | 24 | 4.62 | 0.09 | 0.813 | 0.801 | 0.038* | 11 | 2 | 0.0445 | H3(10)H29(1) | 1 |
| 33 Laotudingzi | 124.90 | 41.33 | 869 | 23 | 4.41 | 0.03 | 0.786 | 0.803 | 0.220 | 12 | 1 | 0.0000 | H3(12) | 0 |
| 34 Renaozhen | 126.01 | 41.41 | 933 | 24 | 4.41 | 0.05 | 0.781 | 0.798 | 0.134 | 11 | 1 | 0.0000 | H3(11) | 0 |
| 37 Dasuhe | 125.00 | 41.93 | 474 | 24 | 4.31 | 0.02 | 0.772 | 0.771 | 0.654 | 12 | 1 | 0.0000 | H3(12) | 0 |
| 39 Changbai Mt. | 128.07 | 42.06 | 987 | 24 | 4.24 | 0.04 | 0.784 | 0.657 | 0.000* | 11 | 1 | 0.0000 | H3(11) | 0 |
| 40 Songjianghe | 127.51 | 42.20 | 902 | 24 | 3.81 | 0.06 | 0.721 | 0.729 | 0.147 | 11 | 1 | 0.0000 | H3(11) | 0 |
| 42 Longwan | 126.45 | 42.37 | 781 | 22 | 4.56 | 0.05 | 0.795 | 0.787 | 0.111 | 9 | 1 | 0.0000 | H3(9) | 0 |
| 43 Lushuihe | 127.77 | 42.53 | 668 | 24 | 4.04 | 0.02 | 0.726 | 0.679 | 0.004* | 8 | 1 | 0.0000 | H3(8) | 0 |
| 45 Lianhuashan | 126.24 | 43.19 | 862 | 24 | 3.89 | 0.03 | 0.709 | 0.703 | 0.278 | 12 | 2 | 0.0408 | H3(11)H33(1) | 0 |
| 46 Wangqing | 130.18 | 43.35 | 767 | 23 | 4.41 | 0.06 | 0.785 | 0.819 | 0.760 | 12 | 1 | 0.0000 | H3(12) | 0 |
| 48 Huangnihe | 128.01 | 43.58 | 564 | 24 | 4.31 | 0.03 | 0.792 | 0.762 | 0.190 | 11 | 1 | 0.0000 | H3(11) | 0 |
| 49 Jingpo Lake | 128.54 | 44.18 | 555 | 24 | 4.17 | 0.05 | 0.752 | 0.790 | 0.145 | 12 | 1 | 0.0000 | H3(12) | 0 |
| 50 Fangzheng | 128.99 | 45.67 | 172 | 24 | 4.19 | 0.00 | 0.749 | 0.752 | 0.074 | 11 | 1 | 0.0000 | H3(11) | 0 |
| 51 Qingshan | 131.19 | 46.58 | 403 | 23 | 4.18 | 0.00 | 0.750 | 0.759 | 0.355 | 11 | 2 | 0.0445 | H3(10)H31(1) | 1 |
| 52 Yilan | 129.38 | 46.63 | 572 | 24 | 4.19 | 0.01 | 0.763 | 0.782 | 0.402 | 12 | 1 | 0.0000 | H3(12) | 0 |
| 53 Langxiang | 129.06 | 46.69 | 381 | 24 | 3.99 | 0.02 | 0.723 | 0.790 | 0.692 | 12 | 1 | 0.0000 | H3(12) | 0 |

| **Table S1 continuous** | | | | | | | | | | | | | | |
| --- | --- | --- | --- | --- | --- | --- | --- | --- | --- | --- | --- | --- | --- | --- |
|  |  |  |  | **Nuclear** **Microsatellite** | | | | | | **Chloroplast DNA** | | | | |
| **Region/population** | **Longitude(°E)** | **Latitude(°N)** | **Alt (m)** | ***N_M_*** | ***AR*** | ***PAR*** | ***H_E_*** | ***H_O_*** | ***HW*** | ***N_C_*** | ***H*** | ***π*(10^-3^)** | **Haplotype** | **Private sites** |
| 54 Daliangzi | 129.88 | 46.75 | 488 | 24 | 3.99 | 0.01 | 0.720 | 0.740 | 0.632 | 12 | 1 | 0.0000 | H3(12) | 0 |
| 55 Raohe | 133.58 | 46.83 | 184 | 24 | 4.09 | 0.02 | 0.748 | 0.726 | 0.084 | 12 | 1 | 0.0000 | H3(12) | 0 |
| 56 Liangshui | 128.88 | 47.18 | 582 | 24 | 4.20 | 0.01 | 0.756 | 0.757 | 0.126 | 12 | 1 | 0.0000 | H3(12) | 0 |
| 57 Jinshantun | 129.61 | 47.36 | 247 | 24 | 4.22 | 0.08 | 0.767 | 0.709 | 0.005* | 12 | 1 | 0.0000 | H3(12) | 0 |
| 58 Lingnan | 130.43 | 48.06 | 285 | 23 | 4.21 | 0.02 | 0.755 | 0.759 | 0.054 | 12 | 1 | 0.0000 | H3(12) | 0 |
| 59 Wuying | 129.19 | 48.21 | 305 | 24 | 3.94 | 0.01 | 0.722 | 0.778 | 0.662 | 12 | 1 | 0.0000 | H3(12) | 0 |
| 60 Tangwanghe | 129.87 | 48.59 | 401 | 24 | 3.75 | 0.01 | 0.686 | 0.681 | 0.800 | 10 | 1 | 0.0000 | H3(10) | 0 |
| 61 Wuyiling | 129.65 | 48.73 | 485 | 21 | 3.74 | 0.00 | 0.692 | 0.688 | 0.502 | 12 | 1 | 0.0000 | H3(12) | 0 |
| 62 Shengshan | 126.78 | 49.48 | 443 | 24 | 3.77 | 0.02 | 0.698 | 0.724 | 0.819 | 11 | 1 | 0.0000 | H3(11) | 0 |
| **Korean Peninsula** |  |  |  |  | **4.80** | **1.47** | **0.834** | **0.809** |  |  | **6** | **0.9787** |  | **4** |
| 24 Jiri Mt. | 127.49 | 35.29 | 667 | 22 | 4.79 | 0.12 | 0.820 | 0.831 | 0.141 | 22 | 4 | 1.0759 | H24(9)H25(7)H26(2)H27(4) | 3 |
| 25 Gariwang Mt. | 128.56 | 37.43 | 435 | 22 | 4.91 | 0.13 | 0.838 | 0.813 | 0.250 | 12 | 2 | 0.0740 | H3(10)H28(2) | 0 |
| 26 Sorak Mt. | 128.49 | 38.17 | 305 | 21 | 4.22 | 0.08 | 0.753 | 0.775 | 0.354 | 12 | 2 | 0.0408 | H3(11)H28(1) | 0 |
| **Japan** |  |  |  |  | **--** | **--** | **--** | **--** | **--** |  | **1** | **--** |  | **0** |
| 63 Akagiyama | 139.15 | 36.49 | 1770 | 2 | **--** | **--** | **--** | **--** | **--** | 2 | 1 | **--** | H32(2) | 0 |
| **Total** |  |  |  | **1236** |  |  | **0.739** | **0.74** |  | **731** | **1.9** | **0.1611** |  |  |

| Table S2 The information of chloroplast DNA primers used in this study | | | | | |
| --- | --- | --- | --- | --- | --- |
| **Chloroplast fragment** | **Sequenced length** | **Primers (5'-3')** | | **Annealing temperature**  **(℃)** | **Reference** |
| *psbA*-*trnH* | 389-408 | *trnH^GUG^* | CGCGCATGGTGGATTCACAATCC | 54 |  |
|  |  | *psbA* | GTTATGCATGAACGTAATGCTC |  |  |
| *trnL*-*trnF* | 839-855 | *trnL* | CGAAATCGGTAGACGCTACG | 54 | [Taberlet et al. (1991)](#_ENREF_3) |
|  |  | *trnF* | ATTTGAACTGGTGACACGAG |  |  |
| *rpl16* | 814-854 | *rpL16F* | GCTATGCTTAGTGTGTGACTCGTTG | 52 | [Small et al. (1998)](#_ENREF_2) |
|  |  | *rpL16R* | CCCTTCATTCTTCCTCTATGTTG |  |  |

| Table S2 The information of haplotypes used to estimate the divergence time of *Acer mono* | | | | |
| --- | --- | --- | --- | --- |
|  |  | **Haplotypes** | | |
| **Section** | **Species** | ***psbA***-***trnH*** | ***trnL***-***trnF*** | ***rpl16*** |
| Sect. Platanoidea | *Acer*. *mono* | A8, A9, A10 | B1, B2, B3 | C1, C2 |
| Sect. Ginnala | *A*. *ginnala* | A1 | B6 | C5 |
| Sect. Macrantha | *A*. *caudatifolium* | A2 | B14 | C8 |
| Sect. Macrantha | *A*. *davidii* | A3, A5, A6 | B11, B12, B13 | C3 |
| Sect. Microcarpa | *A*. *oliverianum subsp*. *formosanum* | A7 | B9, B10 | C7 |
| Sect. Microcarpa | *A*. *oliverianum subsp*. *oliverianum* | A1, A5, A7 | B4, B5, B6 | C3, C4, C5 |
| Sect. Palmata | *A*. *palmatum* | A3, A4, A5, A6, A7 | B5, B7, B8, B15 | C6, C7, C9 |
|  | *Dipteronia*. *sinensis* | A11, A12 | B17 | - |
|  | *D*. *dyerana* | A13 | B16 | C10, C11 |

Reference

SANG, T., CRAWFORD, D. & STUESSY, T. 1997. Chloroplast DNA phylogeny, reticulate evolution, and biogeography of Paeonia (Paeoniaceae). *American Journal of Botany,* 84**,** 1120-1120.

SMALL, R. L., RYBURN, J. A., CRONN, R. C., SEELANAN, T. & WENDEL, J. F. 1998. The tortoise and the hare: choosing between noncoding plastome and nuclear Adh sequences for phylogeny reconstruction in a recently diverged plant group. *American Journal of Botany,* 85**,** 1301-1315.

TABERLET, P., GIELLY, L., PAUTOU, G. & BOUVET, J. 1991. Universal primers for amplification of three non-coding regions of chloroplast DNA. *Plant molecular biology,* 17**,** 1105-1109.

TATE, J. A. & SIMPSON, B. B. 2003. Paraphyly of Tarasa (Malvaceae) and diverse origins of the polyploid species. *Systematic Botany,* 28**,** 723-737.

| Table S4 CpDNA haplotypes | | | | | | | | | | | | | | | | | | | | | | | | | | | | |
| --- | --- | --- | --- | --- | --- | --- | --- | --- | --- | --- | --- | --- | --- | --- | --- | --- | --- | --- | --- | --- | --- | --- | --- | --- | --- | --- | --- | --- |
| Haplotype | n | Location of polymorphism sites | | | | | | | | | | | | | | | | | | | | | | | | | | |
|  |  | *psbA*-*trnH* | | | | | | | | | | | | | | | | | | | |  | *trnF*-*trnL* | | | | | |
|  |  | 16 | 30 | 59 | 95 | 104 | 107 | 156 | 166 | 222 | 235 | 271 | 291 | 316 | 333 | 335 | 346 | 354 | 393 | 413 | 429 |  | 482 | 524 | 605 | 652 | 719 | 843 |
| H1 | 71 | C | - | C | I^2^ | T | G | A | A | G | - | - | - | A | C | T | C | T | G | I^8^ | G |  | I^9^ | A | T | A | C | T |
| H2 | 1 | C | - | C | I^2^ | T | G | A | A | G | - | - | - | A | C | T | C | T | G | I^8^ | G |  | I^9^ | A | T | A | C | T |
| H3 | 432 | T | I^1^ | C | I^3^ | G | G | A | G | T | - | I^5^ | - | T | C | T | C | T | T | - | G |  | - | G | T | A | T | C |
| H4 | 15 | T | I^1^ | C | I^3^ | T | G | A | G | T | - | I^5^ | - | T | C | T | C | T | G | - | G |  | - | G | C | A | C | C |
| H5 | 67 | T | I^1^ | C | - | T | G | A | G | T | - | - | - | T | C | T | C | T | G | - | G |  | - | G | T | A | C | C |
| H6 | 5 | T | I^1^ | C | - | T | G | A | G | T | - | - | - | T | C | T | C | T | G | - | G |  | - | G | T | A | C | C |
| H7 | 8 | T | I^1^ | C | I^3^ | T | G | A | G | T | - | - | - | T | C | T | C | T | G | - | G |  | - | G | T | A | C | C |
| H8 | 1 | T | I^1^ | C | - | T | G | A | G | T | - | - | - | T | C | T | C | T | G | - | G |  | - | G | T | A | C | C |
| H9 | 2 | T | I^1^ | C | - | T | G | A | G | T | - | - | - | T | C | T | C | T | G | - | G |  | - | G | T | A | C | C |
| H10 | 1 | T | I^1^ | C | - | T | G | A | G | T | I^4^ | - | - | T | C | T | C | T | G | - | G |  | - | G | T | A | C | C |
| H11 | 1 | T | I^1^ | C | - | T | T | A | G | T | - | - | - | T | C | T | C | T | G | - | G |  | - | G | T | A | C | C |
| H12 | 11 | T | I^1^ | C | I^3^ | T | G | A | G | T | - | I^5^ | - | T | C | T | C | T | G | - | G |  | - | G | C | A | C | C |
| H13 | 1 | T | I^1^ | C | I^3^ | T | G | A | G | T | - | I^5^ | - | T | C | T | C | T | G | - | G |  | - | G | C | A | C | C |
| H14 | 3 | T | I^1^ | C | - | T | G | A | G | T | - | - | - | T | C | T | C | T | G | - | G |  | - | G | T | A | T | C |
| H15 | 2 | T | I^1^ | T | - | T | G | A | G | T | - | - | - | T | C | T | C | T | G | - | G |  | - | G | T | A | C | C |
| H16 | 4 | T | I^1^ | T | - | T | G | A | G | T | - | - | - | T | C | T | C | T | G | - | G |  | - | G | T | T | C | C |
| H17 | 1 | T | I^1^ | C | - | T | G | A | G | T | - | - | - | T | C | T | C | T | G | - | G |  | - | G | T | T | C | C |
| H18 | 7 | T | I^1^ | C | I^3^ | G | G | A | G | T | - | I^5^ | - | T | C | T | C | T | T | - | G |  | - | G | T | A | C | C |
| H19 | 21 | T | I^1^ | C | - | T | G | A | G | T | - | - | - | T | C | T | C | T | G | - | G |  | - | G | T | A | C | C |
| H20 | 33 | T | I^1^ | C | I^3^ | T | G | A | G | T | - | I^5^ | - | T | C | T | C | T | G | - | G |  | - | G | T | A | C | C |
| H21 | 1 | T | I^1^ | C | - | T | G | A | G | T | - | - | - | T | C | G | C | T | G | - | G |  | - | G | T | A | C | C |
| H22 | 3 | T | I^1^ | C | I^3^ | T | G | A | G | T | - | I^5^ | - | T | C | T | C | T | G | - | G |  | - | G | T | A | C | C |
| H23 | 10 | T | I^1^ | C | I^3^ | G | G | A | G | T | - | I^5^ | - | T | C | T | T | T | T | - | G |  | - | G | T | A | T | C |
| H24 | 9 | T | I^1^ | C | I^3^ | G | G | A | G | T | - | I^5^ | - | T | C | T | C | T | G | - | G |  | - | G | T | A | T | C |
| H25 | 7 | T | I^1^ | C | I^3^ | T | G | A | G | T | - | I^5^ | - | T | C | T | C | T | G | - | G |  | - | G | T | A | T | C |
| H26 | 2 | T | I^1^ | C | - | T | G | A | G | T | - | - | I^7^ | T | C | T | C | T | G | - | G |  | - | G | T | A | C | C |
| H27 | 4 | T | I^1^ | C | - | T | G | A | G | T | - | - | I^7^ | T | T | T | C | T | G | - | G |  | - | G | T | A | C | C |
| H28 | 3 | T | I^1^ | C | I^3^ | G | G | G | G | T | - | I^5^ | - | T | C | T | C | T | T | - | G |  | - | G | T | A | T | C |
| H29 | 1 | T | I^1^ | C | I^3^ | G | G | A | G | T | - | I^5^ | - | T | C | T | C | T | T | - | G |  | - | G | T | A | T | C |
| H30 | 1 | T | I^1^ | C | I^3^ | G | G | A | G | T | - | I^5^ | - | T | C | T | C | C | T | - | G |  | - | G | T | A | T | C |
| H31 | 1 | T | I^1^ | C | I^3^ | G | G | A | G | T | - | I^5^ | - | T | C | T | C | T | T | - | C |  | - | G | T | A | T | C |
| H32 | 2 | T | I^1^ | C | - | T | G | A | G | T | - | - | - | T | C | T | C | T | G | - | G |  | - | G | T | A | C | C |
| H33 | 1 | T | I^1^ | C | I^3^ | G | G | A | G | T | - | I^6^ | - | T | C | T | C | T | T | - | G |  | - | G | T | A | T | C |

n, Number of indviduals; I^x^, insertion(‘x’ is the number of bases inserted)

| Table S4 continuous | | | | | | | | | | | | | | | | | | | | | | | | | | | | | | |
| --- | --- | --- | --- | --- | --- | --- | --- | --- | --- | --- | --- | --- | --- | --- | --- | --- | --- | --- | --- | --- | --- | --- | --- | --- | --- | --- | --- | --- | --- | --- |
| Haplotype | n | Location of polymorphism sites | | | | | | | | | | | | | | | | | | | | | | | | | | | | |
|  |  | *trnL*-*trnF* | | | | | | | | | | | | | | |  | *rpl16* | | | | | | | | | | | | |
|  |  | 900 | 920 | 961 | 1049 | 1075 | 1078 | 1241 | 1264 | 1286 | 1305 | 1402 | 1622 | 1678 | 1803 | 1820 |  | 1844 | 1853 | 1864 | 1885 | 1918 | 1945 | 1947 | 1988 | 1995 | 1999 | 2002 | 2027 | 2097 |
| H1 | 71 | C | T | C | T | G | I^10^ | C | A | T | G | G | T | C | G | G |  | T | - | G | G | I^12^ | G | C | T | A | G | G | A | T |
| H2 | 1 | C | T | C | T | G | I^10^ | C | A | T | G | G | T | C | G | G |  | T | - | G | G | I^12^ | G | C | T | A | G | G | G | T |
| H3 | 432 | A | T | C | T | A | - | C | A | T | A | T | T | C | G | A |  | C | - | T | A | I^13^ | G | C | G | G | A | A | A | G |
| H4 | 15 | A | T | C | G | A | - | C | A | T | A | T | T | C | G | A |  | C | - | T | A | I^13^ | G | C | G | G | A | A | A | G |
| H5 | 67 | A | C | C | T | A | - | C | A | T | A | T | T | C | G | A |  | C | - | T | A | I^13^ | G | C | G | G | A | A | A | G |
| H6 | 5 | A | C | C | T | A | - | C | G | T | A | T | T | C | G | A |  | C | - | T | A | - | G | C | G | G | A | A | A | G |
| H7 | 8 | A | T | C | T | A | - | C | A | T | A | T | T | C | G | A |  | C | - | T | A | I^13^ | G | C | G | G | A | A | A | G |
| H8 | 1 | A | T | C | T | A | - | C | A | T | A | T | T | C | G | A |  | C | - | T | A | I^13^ | A | C | G | G | A | A | A | G |
| H9 | 2 | A | C | T | T | A | - | C | A | T | A | T | T | C | G | A |  | C | - | T | A | I^13^ | G | C | G | G | A | A | A | G |
| H10 | 1 | A | C | C | T | A | - | C | A | T | A | T | T | C | G | A |  | C | - | T | A | I^13^ | G | C | G | G | A | A | A | G |
| H11 | 1 | A | T | C | T | A | - | C | A | T | A | T | T | C | G | A |  | C | - | T | A | I^13^ | G | T | G | G | A | A | A | G |
| H12 | 11 | A | T | C | G | A | - | C | A | T | A | T | T | C | G | A |  | C | - | T | A | - | G | C | G | G | A | A | A | G |
| H13 | 1 | A | T | C | G | A | - | C | A | T | A | T | T | C | T | A |  | C | - | T | A | I^13^ | G | C | G | G | A | A | A | G |
| H14 | 3 | A | T | C | T | A | - | C | A | T | A | T | T | C | G | A |  | C | - | T | A | I^13^ | G | C | G | G | A | A | A | G |
| H15 | 2 | A | C | C | T | A | - | C | A | T | A | T | T | T | G | A |  | C | - | T | A | I^13^ | G | C | G | G | A | A | A | G |
| H16 | 4 | A | C | C | T | A | - | C | A | T | A | T | T | T | G | A |  | C | - | T | A | I^13^ | G | C | G | G | A | A | A | G |
| H17 | 1 | A | C | C | T | A | - | C | A | T | A | T | T | T | G | A |  | C | - | T | A | I^13^ | G | C | G | G | A | A | A | G |
| H18 | 7 | A | C | C | T | A | - | C | A | T | A | T | T | C | G | A |  | C | - | T | A | I^13^ | G | C | G | G | A | A | A | G |
| H19 | 21 | A | C | C | T | A | - | C | A | T | A | T | T | C | G | A |  | C | I^11^ | T | A | I^13^ | G | C | G | G | A | A | A | G |
| H20 | 33 | A | T | C | T | A | - | C | A | T | A | T | T | C | G | A |  | C | - | T | A | I^13^ | G | C | G | G | A | A | A | G |
| H21 | 1 | A | C | C | T | A | - | C | A | T | A | T | T | C | G | A |  | C | I^11^ | T | A | I^13^ | G | C | G | G | A | A | A | G |
| H22 | 3 | A | T | C | T | A | - | C | A | T | A | T | C | C | G | A |  | C | - | T | A | I^13^ | G | C | G | G | A | A | A | G |
| H23 | 10 | A | T | C | T | A | - | C | A | T | A | T | T | C | G | A |  | C | - | T | A | I^13^ | G | C | G | G | A | A | A | G |
| H24 | 9 | A | T | C | T | A | - | C | A | T | A | T | T | C | G | A |  | C | - | T | A | I^13^ | G | C | G | G | A | A | A | G |
| H25 | 7 | A | T | C | T | A | - | T | A | T | A | T | T | C | G | A |  | C | - | T | A | I^13^ | G | C | G | G | A | A | A | G |
| H26 | 2 | A | T | C | T | A | - | C | A | T | A | T | T | C | G | A |  | C | - | T | A | I^13^ | G | C | G | G | A | A | A | G |
| H27 | 4 | A | T | C | T | A | - | C | A | T | A | T | T | C | G | A |  | C | - | T | A | I^13^ | G | C | G | G | A | A | A | G |
| H28 | 3 | A | T | C | T | A | - | C | A | T | A | T | T | C | G | A |  | C | - | T | A | I^13^ | G | C | G | G | A | A | A | G |
| H29 | 1 | A | T | C | T | A | - | C | A | G | A | T | T | C | G | A |  | C | - | T | A | I^13^ | G | C | G | G | A | A | A | G |
| H30 | 1 | A | T | C | T | A | - | C | A | T | A | T | T | C | G | A |  | C | - | T | A | I^13^ | G | C | G | G | A | A | A | G |
| H31 | 1 | A | T | C | T | A | - | C | A | T | A | T | T | C | G | A |  | C | - | T | A | I^13^ | G | C | G | G | A | A | A | G |
| H32 | 2 | A | T | C | T | A | - | C | A | T | A | T | T | C | G | A |  | C | - | T | A | I^13^ | G | C | G | G | A | A | A | G |
| H33 | 1 | A | T | C | T | A | - | C | A | T | A | T | T | C | G | A |  | C | - | T | A | I^13^ | G | C | G | G | A | A | A | G |

| Table S3 Results of analysis of molecular variance (AMOVA) of cpDNA haplotypes and nuclear microsatellite alleles frequencies for populations and population groups of *Acer mono* | | | | | | | |
| --- | --- | --- | --- | --- | --- | --- | --- |
| Source of variation | | CpDNA | | |  | Nuclear Microsatellite | |
|  |  | Percentage of variation | *P* value | *Gst* |  | Percentage of variation | *P* value |
| Whole | Among regions | 45.70 | 0.000 |  |  | 4 | 0.01 |
|  | Among populations within region | 49.99 | 0.000 | 0.822 |  | 8 | 0.01 |
|  | within populations | 4.30 |  |  |  | 84 |  |
| II | Among populations | 0.01 | 0.445 | -0.004 |  | 3 | 0.01 |
|  | Within populations | 99.99 |  |  |  | 97 |  |
| III | Among populations | 92.38 | 0.000 | 0.872 |  | 12 | 0.01 |
|  | Within populations | 7.62 |  |  |  | 88 |  |
| IV | Among regions | 90.75 | 0.002 |  |  | 7 | 0.01 |
|  | Among populations within region | 6.23 | 0.000 | 0.715 |  | 16 | 0.01 |
|  | within populations | 3.03 |  |  |  | 77 |  |
| KP | Among populations | 45.71 |  | 0.463 |  | 3 | 0.01 |
|  | within populations | 54.29 |  |  |  | 97 |  |
| *G*ST, interpopulation differentiation | | | | | | | |

| Table S6 The SAMOVA of *Acer mono* based on cpDNA | | |
| --- | --- | --- |
| ***K*** | ***F_CT_*** | **groups** |
| **2** | 0.914 | (1-4)(5-7,9,11,13-62) |
| **3** | 0.922 | (1-4)(6,7,11,13-15,18,21)(5,9,16,17,19,20,22-62) |
| **4** | 0.944 | (1-4)(6,7,11,13-15,18,21)(5,9,16,17,20)(19,22-62) |
| **5** | 0.954 | (1-4)(6,7,11,13-15,18,21)(5,9)(16,17,20,24)(19,22,23,25-62) |
| **6** | 0.958 | (1-4)(6,11,13-15,18,21)(5,9)(7,16,17,20)(24)(19,22,23,25-62) |
| **7** | 0.962 | (1-4)(6,11,13-15)(18,21)(5,9)(7,16,17,20)(24)(19,22,23,25-62) |
| **8** | 0.964 | (1-4)(6,11,13-15)(18,21)(5,9)(7)(16,17,20)(24)(19,22,23,25-62) |
| **9** | 0.964 | (1-4)(6,11,13-15)(18)(21)(5,9)(7)(16,17,20)(24)(19,22,23,25-62) |
| **10** | 0.965 | (1-4)(6,11,13-15)(18)(21)(5,9)(7)(16,17,20)(24)(23)(19,22,25-62) |
| **11** | **0.966** | **(1-4)(6**,**11**,**13-15)(18)(21)(5**,**9)(7)(16**,**17**,**20)(24)(23)(32)(19**,**22**,**25-31**,**33-62)** |
| **12** | 0.962 | (1-4)(6,7,11,13-15)(18,21)(5)(9)(16,17,20)(24)(23)(32)(22)(29)(19,25-28,30-31,33-62) |
| **13** | 0.964 | (1-4)(6,11,13-15)(18)(21)(5,9)(7)(16,17,20)(24)(23)(32)(25)(60)(19,22,26-31,33-59,61,62) |
| *K*, number of groups; *F_CT_*, correlation of haplotypes within groups relative to total；groups, populations in groups | | |

Figure legends

Fig. S1 *LnP*(*D*) and Δ*K* for each *K* in the STRUCTURE analysis
